# Reduced *LINC01089* Expression Impairs Coordinated Heme–Globin Transcriptional Programs in Human Erythroid Cells

**DOI:** 10.3390/ijms27104394

**Published:** 2026-05-14

**Authors:** Yuan Xue, Xiaohong Li, Yonggang Zhang, Feng Ma

**Affiliations:** Center of Stem Cell and Regenerative Medicine, Institute of Blood Transfusion, Chinese Academy of Medical Sciences & Peking Union Medical College (CAMS & PUMC), Chengdu 610052, China; yue_xue0805@126.com (Y.X.); xhli_0423@163.com (X.L.)

**Keywords:** *LINC01089*, lncRNA, erythroid maturation, hemoglobin

## Abstract

Long non-coding RNAs (lncRNAs) have emerged as important regulators of developmental processes. Recent studies have established roles for lncRNAs in human and murine erythroid regulation, yet additional regulators remain to be discovered. To identify lncRNA candidates involved in human erythroid regulation, we established a pooled genome-editing screen strategy using human embryonic stem cells (hESCs). Long Intergenic Non-Protein Coding RNA 1089 (*LINC01089*) was selected for functional investigation. We found that reduced *LINC01089* expression impaired erythroid differentiation. Transcriptomic profiling revealed consistent downregulation of genes related to hemoglobin assembly, heme biosynthesis, and membrane maturation, suggesting that *LINC01089* supports coordinated erythroid transcriptional programs. In particular, progressive reduction of *HBB* expression emerged as a key transcriptional anchor. Enrichment analyses of upregulated genes identified recurrent focal adhesion signatures, suggesting a potential link between *LINC01089* and focal adhesion kinase (FAK)-related signaling. Given prior evidence linking *LINC01089* to FAK phosphorylation, we performed a pilot FAK-inhibition experiment, producing a partial shift toward wild-type *HBB* expression and supporting FAK/phosphorylated FAK (pFAK) signaling as a potential contributing axis in the impaired transcriptional programs. Together, our findings identify *LINC01089* as a novel lncRNA linked to coordinated heme–globin transcriptional programs in human erythroid differentiation, with possible involvement of the FAK/pFAK axis.

## 1. Introduction

The regulatory framework of erythropoiesis has classically centered on transcription factors (TFs) and regulatory proteins that govern cell fate decisions and functional maturation [1,2]. Over time, this framework has expanded into a broader network, involving epigenetic modifications, post-transcriptional control, and other context-dependent mechanisms [3,4,5]. Erythropoiesis is now understood as a fine-tuned process with multiple regulatory layers supporting cell-lineage commitment, differentiation, and terminal maturation [6]. Disruption of erythropoiesis contributes to a range of erythroid disorders [6,7]. Understanding the molecular mechanisms underlying erythroid development benefits fundamental biology and elucidates pathological erythroid abnormalities.

Long non-coding RNAs (lncRNAs), transcripts over 200 nucleotides, have been reported to play regulatory roles in erythroid survival, globin regulation, heme biosynthesis, chromatin configuration, and maturation [8]. For example, *lincRNA-EPS* supports survival during murine terminal erythroid differentiation [9]. Further, *UCA1* stabilizes *ALAS2* messenger RNA (mRNA) via PTBP1 to sustain heme biosynthesis in human erythropoiesis, while *GATA2-AS1* (*GATA2AS*) has recently been reported to bind to LRF and KLF1, influencing their genome-wide DNA binding activities [10,11].

To date, most erythroid lncRNA candidates have been identified through bulk or single-cell transcriptomic profiling across developmental stages or purified erythroid subpopulations, often combined with chromatin accessibility, transcription factor occupancy, or enhancer states [8,12]. However, some lncRNAs that act transiently or lack obvious mechanistic anchors may escape nomination. Pooled clustered regularly interspaced short palindromic repeats (CRISPR) screening provides a complementary route, enabling nomination based on functional output rather than expression dynamics or predefined molecular anchors. Paired-guide RNA (pgRNA) deletion may be more effective in generating loss-of-function perturbations than single-guide RNA (sgRNA) designs for lncRNA loci [13]. Although a genome-scale sgRNA screen for protein-coding genes (PCGs) in erythroid differentiation has recently been reported [14], erythroid-focused pooled lncRNA screening remains comparatively limited.

Based on these considerations, we established a tailored pooled pgRNA screening strategy for lncRNA nomination during human erythroid differentiation. Using a co-culture system of human embryonic stem cells (hESCs) and mouse aorta-gonad-mesonephros-derived stromal cell line S3 (AGM-S3) cells [15,16], we identified Long Intergenic Non-Protein Coding RNA 1089 (*LINC01089*) as a candidate for functional validation.

*LINC01089* is located on chromosome 12q24.31. Existing studies of *LINC01089* have focused predominantly on cancer biology, where it has been implicated in the regulation of cancer cell migration, invasion, and signaling [17]. Outside tumor-related contexts, only limited evidence has been reported, including roles in mesenchymal stem cell osteogenic differentiation and endothelial repair [18,19]. However, its role in erythroid development remains unknown.

In this study, we investigated the role of *LINC01089* in human erythroid differentiation using in vitro culture models. Combined phenotypic and transcriptomic analyses showed that reduced *LINC01089* expression led to multifaceted defects during erythroid differentiation, with impaired establishment of coordinated heme–globin transcriptional programs. Recurrent enrichment of focal adhesion-related terms emerged in our transcriptomic data, suggesting a potential connection with focal adhesion kinase (FAK), a central mediator of focal adhesion signaling [20]. Given prior evidence that *LINC01089* can bind FAK and suppress its phosphorylation [21], we performed a pilot FAK-inhibition experiment, supporting the FAK/phosphorylated FAK (pFAK) axis as a potential contributor to the erythroid abnormalities. This study identifies *LINC01089* as a previously unrecognized lncRNA associated with human erythroid differentiation and provides a foundation for elucidating its mechanistic basis.

## 2. Results

### 2.1. A pgRNA CRISPR/Cas9 Screen Nominated LINC01089 for Erythroid Functional Validation

**Figure 1:**
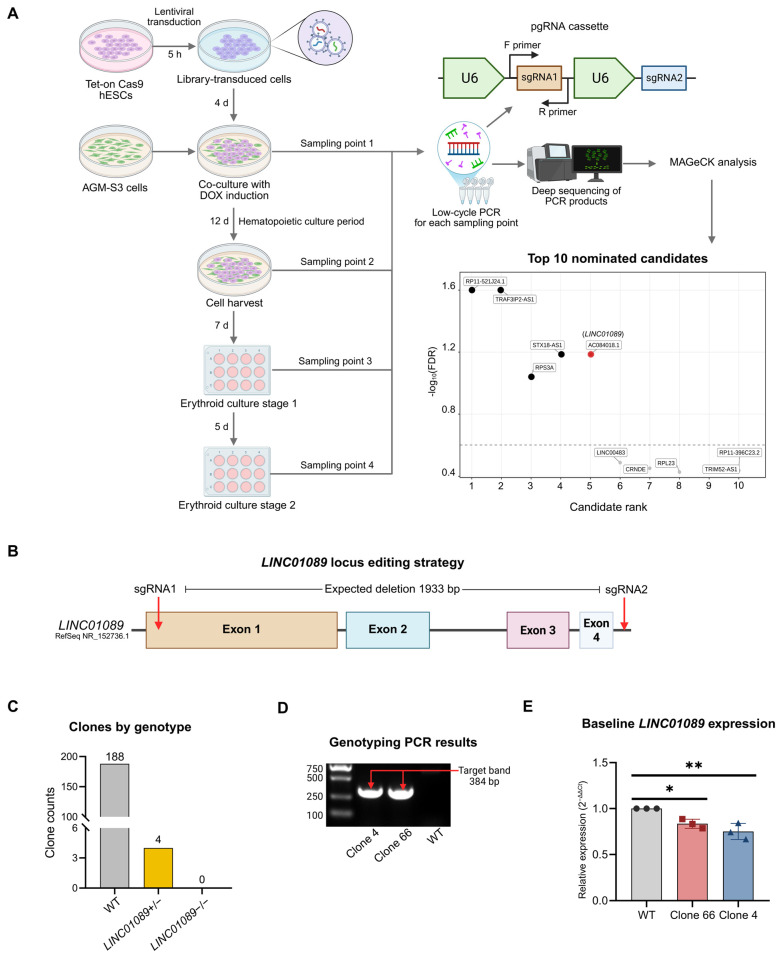
Pooled paired-guide RNA (pgRNA) screen and the generation of Long Intergenic Non-Protein Coding RNA 1089 (*LINC01089*)+/− human embryonic stem cell (hESC) clones. (**A**) Schematic of the pooled pgRNA screening design. A Tet-On CRISPR-associated protein 9 (Cas9) hESC line was used for aorta-gonad-mesonephros-derived stromal cell line S3 (AGM-S3) co-culture followed by erythroid differentiation. pgRNA abundance was profiled at multiple sampling points by deep sequencing and analyzed using Model-based Analysis of Genome-wide CRISPR-Cas9 Knockout (MAGeCK) for candidate ranking. The lower right panel shows the top 10 nominated candidates from the comparison between sampling point 4 and sampling point 3 in the positive-selection direction. *AC084018.1* (*LINC01089*) is highlighted in red. The pgRNA library information and MAGeCK results are provided in Supplementary Datasets S1 and S2. (**B**) Schematic of the clustered regularly interspaced short palindromic repeats/Cas9 (CRISPR/Cas9) editing strategy targeting the *LINC01089* locus. A representative transcript model is shown. Two single-guide RNAs (sgRNAs) were designed to generate a 1933-base pair (bp) sequence deletion. CRISPOR (https://crispor.org; accessed on 7 May 2026) off-target prediction did not suggest a broad high-risk off-target profile for the two sgRNAs (Supplementary Dataset S3). (**C**) Summary of clone genotyping results after CRISPR/Cas9 editing, showing the numbers of wild-type (WT), *LINC01089*+/−, and *LINC01089*−/− clones. (**D**) Representative genotyping polymerase chain reaction (PCR) results for two independent *LINC01089*+/− clones (Clone 4 and Clone 66) and WT cells. The expected deletion band is indicated. The original uncropped gel image is provided in Supplementary Dataset S4. (**E**) Baseline *LINC01089* expression in WT and *LINC01089*+/− clones at the undifferentiated hESC stage. Both clones showed lower *LINC01089* expression relative to WT. * *p* < 0.05; ** *p* < 0.01.

To nominate lncRNA candidates for functional validation, we performed a pooled CRISPR/CRISPR-associated protein 9 (Cas9) pgRNA screen in an AGM-S3 co-culture erythroid differentiation model (Figure 1A).

Model-based Analysis of Genome-wide CRISPR-Cas9 Knockout (MAGeCK) quality control (QC) analysis indicated that, at baseline input, the library representation was maintained with a 2% zero-count rate (Table S1). A Gini index of 0.132 suggested even distribution of the guide pairs. Importantly, over 96% of targeted genes retained support from ≥2 guide pairs in the erythroid readouts, supporting candidate calling for subsequent analysis (Table S2).

*LINC01089* was nominated by the screen as the only lncRNA candidate passing the false discovery rate (FDR) threshold and read-count quality control among the top-ranked candidates (Figure 1A and Figure S1A). We next designed a CRISPR/Cas9 deletion strategy targeting the *LINC01089* region spanning exons 1–4 to assess the gene more thoroughly (Figure 1B and Figure S1B). Clone genotyping results showed successful deletion of the targeted *LINC01089* genomic segment in 4 of 196 clones (Figure 1C,D). The selected Clone 66 and Clone 4 retained wild-type (WT)-sized amplicons, consistent with heterozygous deletion (Supplementary Dataset S4).

Sanger sequencing further confirmed the edited CRISPR/Cas9 deletion junctions in both clones. The small clone-specific junctional insertions were consistent with end-joining repair after dual-sgRNA cleavage (Figure S1C). However, homozygous knockout clones were not obtained. Reduced *LINC01089* expression was detected at the hESC stage (Figure 1E).

### 2.2. LINC01089+/− Clones Exhibit Impaired Erythroid Output and Altered Erythroid Phenotypes In Vitro

**Figure 2:**
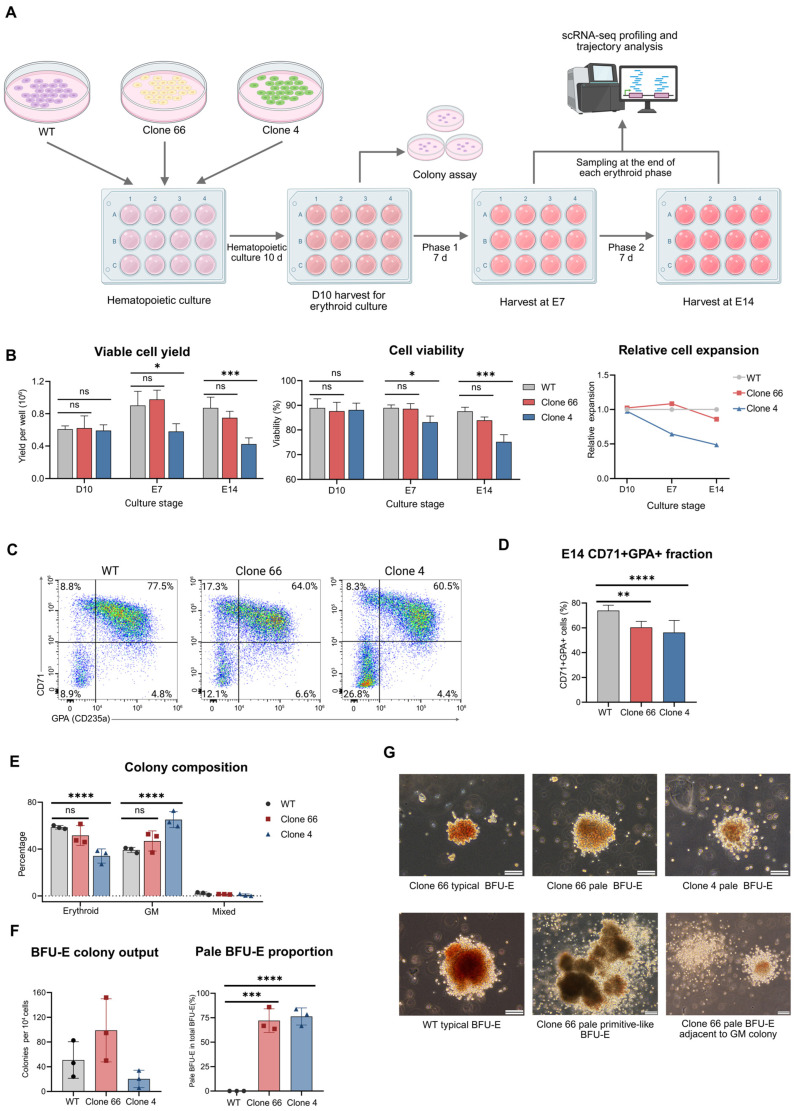
*LINC01089*+/− clones exhibit impaired erythroid output and altered erythroid phenotypes in vitro. (**A**) Schematic of the erythroid differentiation workflow used for *LINC01089* functional validation. WT hESCs and two *LINC01089*+/− clones (Clone 66 and Clone 4) were subjected to hematopoietic induction followed by erythroid culture using the STEMdiff Erythroid Kit. The indicated harvest stages were defined as follows: D10, the end of the hematopoietic culture period (day 10 of culture); E7, the end of phase 1 erythroid culture (day 17 of culture); and E14, the end of phase 2 erythroid culture (day 24 of culture). Samples were collected at the indicated stages for downstream analyses. (**B**) Quantification of viable cell yield, cell viability, and relative live-cell expansion across the indicated culture stages (n = 5). Clone 4 showed reduced expansion and viability at E14, whereas Clone 66 showed a similar trend without reaching statistical significance. * *p* < 0.05; *** *p* < 0.001; ns, not significant. (**C**) Representative flow cytometry plots showing CD71 and glycophorin A (GPA/CD235a) staining at E14 in WT, Clone 66, and Clone 4 cultures. (**D**) Quantification of the CD71+GPA+ fraction at E14 (n = 8), showing reduced CD71+GPA+ fractions in both *LINC01089*+/− clones. ** *p* < 0.01; **** *p* < 0.0001. (**E**) Colony composition analysis showing the proportions of erythroid, granulocyte-macrophage (GM), and mixed colonies (n = 3). Both clones showed a trend toward lower erythroid colony proportions, which reached statistical significance only in Clone 4. A complete colony assay summary is provided in Supplementary Dataset S5. **** *p* < 0.0001; ns, not significant. (**F**) Quantification of burst-forming unit-erythroid (BFU-E) colony output and the proportion of pale BFU-E colonies among total BFU-E colonies (n = 3). BFU-E colony output displayed clone-specific trends, but was not significantly altered, whereas pale BFU-E colonies were selectively observed in the *LINC01089*+/− clones. *** *p* < 0.001; **** *p* < 0.0001. (**G**) Representative images of typical and pale BFU-E colonies from WT and *LINC01089*+/− samples 14 days after methylcellulose seeding. Scale bars, 100 μm.

To investigate the phenotypic impact of heterozygous *LINC01089* deletion, WT hESCs, Clone 66, and Clone 4 were subjected to erythroid culture using the STEMdiff Erythroid Kit (STEMCELL Technologies Canada Inc., Vancouver, BC, Canada) (Figure 2A). Hereafter, E7 and E14 refer to days 7 and 14 of erythroid culture, respectively. Hematopoietic culture yielded comparable cell output and CD34+CD43+ fractions (Figure 2B and Figure S2A). During erythroid culture, both *LINC01089*+/− clones exhibited reduced expansion and clone-dependent viability defects at E14. Flow cytometry analysis further revealed a decreased CD71+glycophorin A (GPA/CD235a)+ fraction in the *LINC01089*+/− cultures at E14 (Figure 2C,D), while the fraction changes at E7 remained clone-specific (Figure S2B). These data support reduced erythroid output at the later phase of erythroid culture.

Total colony composition showed a trend toward reduced erythroid colony proportions in both clones (Figure 2E). Both burst-forming unit-erythroid (BFU-E) and colony-forming unit-erythroid (CFU-E) colony outputs showed clone-specific trends compared to WT (Figure 2F and Figure S2C). Within the erythroid colonies, both clones showed a slight shift in BFU-E and CFU-E ratios (Figure S2D). Notably, fractions of pale BFU-E colonies were detected only in the *LINC01089*+/− clones with comparable proportions (Figure 2F,G and Figure S2E). A total of 10 manually picked pale colonies from both clones in a single batch remained mostly GPA+ by flow cytometry (Figure S2F), supporting their erythroid identity.

### 2.3. Single-Cell RNA Sequencing Reveals Stage-Skewed Distribution of Erythroid Subcluster States in LINC01089+/− Clones

**Figure 3:**
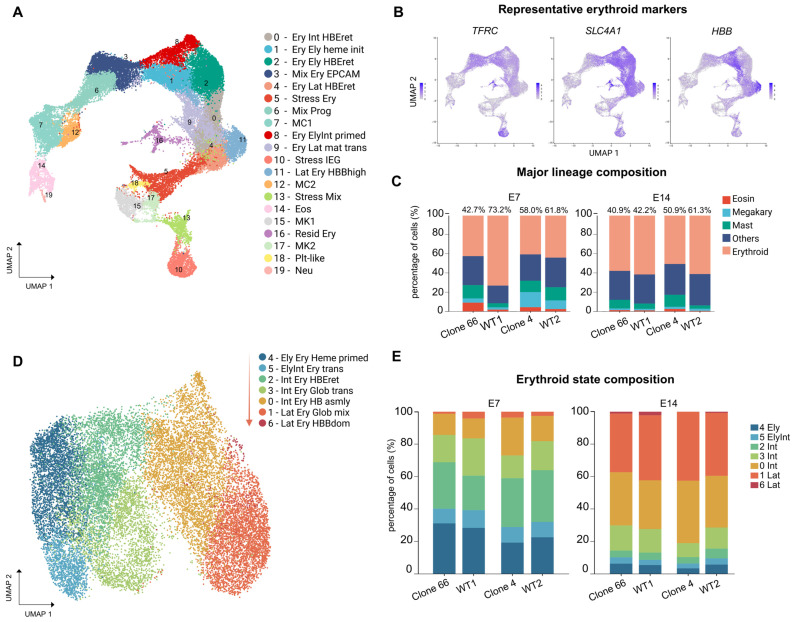
Single-cell RNA sequencing (scRNA-seq) reveals stage-skewed erythroid-state distribution in *LINC01089*+/− clones. (**A**) Uniform manifold approximation and projection (UMAP) visualization of the scRNA-seq data, showing the major cell populations identified across WT and *LINC01089*+/− samples. Abbreviations: Ery, erythroid; Ely, early; Int, intermediate; Lat, late; HBEret, HBE-retained; IEG, immediate-early gene; Resid_Ery, residual erythroid. (**B**) Representative feature plots of erythroid marker genes supporting lineage annotation. (**C**) Major-lineage composition analysis at E7 and E14. Major lineages include erythroid, mast, megakaryocytic, and eosinophilic populations, with the remaining cells grouped as others. (**D**) UMAP of the erythroid compartment after re-clustering, resolving seven erythroid subclusters. The red arrow indicates the direction toward later erythroid subcluster states within this in vitro erythroblast culture model. Abbreviations: Glob_trans, globin transition; Hb_asmly, hemoglobin assembly; Glob_mix, mixed globin state; HBBdom, *HBB*-dominant. (**E**) Composition analysis of erythroid subcluster states at E7 and E14. Clone 66 showed increased representation of earlier and intermediate subcluster states, while Clone 4 showed relative enrichment of later erythroid subcluster states. Cluster colors reflect erythroid subcluster-state progression, with earlier states shown in cooler tones and later subcluster states shown in warmer tones.

To explore how these defects were reflected across the erythroid differentiation landscape, we analyzed WT and *LINC01089*+/− cultures by single-cell RNA sequencing (scRNA-seq) in two separate batches for each clone (Figure 2A). WT and *LINC01089*+/− cells were intermingled across the shared embedding, indicating that the major population structure was preserved at the global level (Figure 3A and Figure S3A).

Major populations were annotated using canonical marker genes (Figure 3B and Figure S3B). Major-lineage composition analysis identified the erythroid compartment (clusters 0, 1, 2, 4, 8, 9, and 11) as the principal lineage altered in *LINC01089*+/− cultures at E14 (Figure 3C), consistent with previous flow cytometry observations.

Further subclustering analysis resolved the erythroid compartment into early heme-primed, intermediate membrane or globin-transition, and late *HBB*-dominant subclusters (Figure 3D and Figure S3C,D). These annotations are interpreted within the erythroblast window of the STEMdiff Erythroid Kit rather than terminal maturation stages.

Composition results showed higher proportions of earlier and intermediate clusters in Clone 66 at both E7 and E14. In contrast, Clone 4 showed relative enrichment of later erythroid subcluster states, which may partly reflect its stronger defects in cell viability and expansion (Figure 3E).

### 2.4. scRNA Analyses Identify Attenuated Hemoglobin- and Erythroid Maturation-Associated Programs in LINC01089+/− Cells

**Figure 4:**
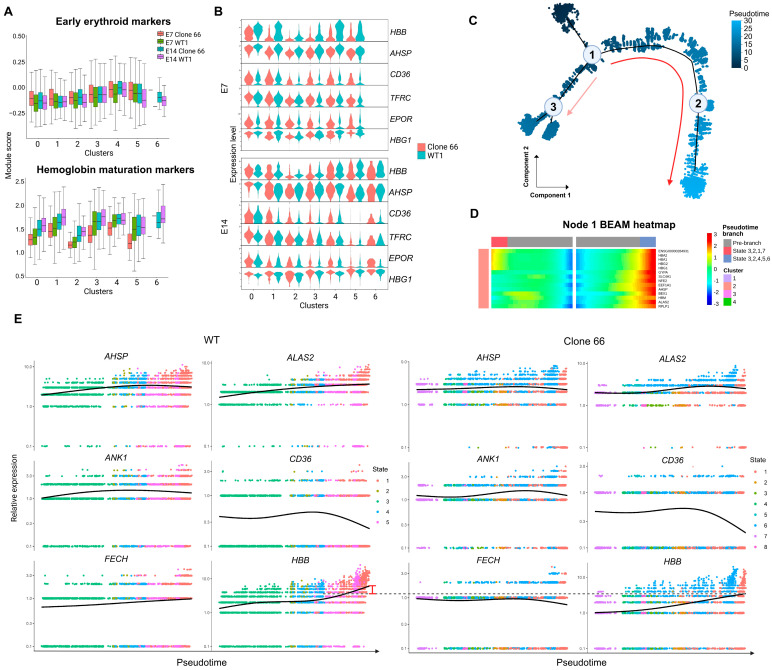
Single-cell program scoring and pseudotime analysis identify attenuated erythroid maturation-associated programs toward late pseudotime states in *LINC01089*+/− cells. (**A**) Module scoring of representative early erythroid markers and hemoglobin maturation markers at E7 and E14 in WT and Clone 66 subclusters. (**B**) Violin plots showing the expression of key erythroid genes across WT1 and Clone 66 subclusters. *HBB* and *AHSP*, markers associated with hemoglobin assembly, were reduced, particularly toward later subclusters. Early erythroid markers *EPOR*, *TFRC*, and *CD36* were not uniformly suppressed. The gene sets used for scoring are provided in Supplementary Dataset S6. (**C**) Pseudotime trajectory reconstructed from erythroid subclusters in all samples, showing the continuous progression path and branch structure. Cells are colored by pseudotime, and major nodes are indicated. The red arrow indicates the main inferred pseudotime progression, whereas the pink arrow indicates an alternative branch with weaker maturation-associated signatures. (**D**) Representative gene cluster from the Node 1 Branch Expression Analysis Modeling (BEAM) heatmap, showing branch-dependent gene-expression patterns along the erythroid trajectory. The branch toward node 3 showed weaker activation of erythroid membrane- and hemoglobin-related genes. (**E**) Pseudotime reconstruction of WT and Clone 66 erythroid cells without batch correction. Representative expression trends of erythroid maturation-related genes along pseudotime are shown. The red bar indicates the relative difference in the *HBB* expression trend between WT and Clone 66.

We next examined gene-program activity by module scoring. The hemoglobin maturation module showed a progressive reduction in *LINC01089*+/− clones from earlier to later erythroid subclusters (Figure 4A and Figure S4A). The early erythroid module showed patterns consistent with the subcluster composition tendencies of each clone. Together, these patterns suggested that the major transcriptional difference was an imbalanced establishment of hemoglobin maturation-related programs at later erythroid subcluster states, accompanied by clone-specific stage biases. This interpretation was also supported by representative genes in Clone 66 (Figure 4B).

Pseudotime analysis further placed erythroid cells from all samples into a coherent erythroid pseudotime continuum (Figure 4C and Figure S4B). Branch Expression Analysis Modeling (BEAM) results at node 1 showed branch-dependent expression patterns of erythroid genes. One branch was enriched for fetal/embryonic hemoglobin features and the other for membrane and heme maturation signals (Figure 4D and Figure S4C), consistent with previous reports describing the globin profiles in hESC-derived erythroid cultures [22]. Mapping of WT subclusters onto the trajectory was consistent with their annotations. Clusters 4, 0, 1, and 6 represented anchors for early, intermediate, and late erythroid subcluster states, while clusters 5, 2, and 3 occupied intermediate positions between clusters 4 and 0 (Figure S4D).

To better capture transcriptional dynamics, we performed sample-specific pseudotime reconstruction separately in WT and Clone 66 cells without batch correction (Figure S4E). In WT cells, branch-dependent analysis revealed coordinated induction of maturation-associated genes along pseudotime, including *AHSP*, *ALAS2*, *FECH*, *SLC4A1*, and *HBB*. In Clone 66 cells, these trends were blunted, delayed, or reversed toward late pseudotime positions, suggesting attenuated establishment of erythroid maturation-associated programs (Figure 4E and Figure S4F).

### 2.5. Bulk Transcriptomics Identifies Shared Erythroid Transcriptional Defects and Reduced Coordination of Heme–Globin Programs in LINC01089+/− Clones

**Figure 5:**
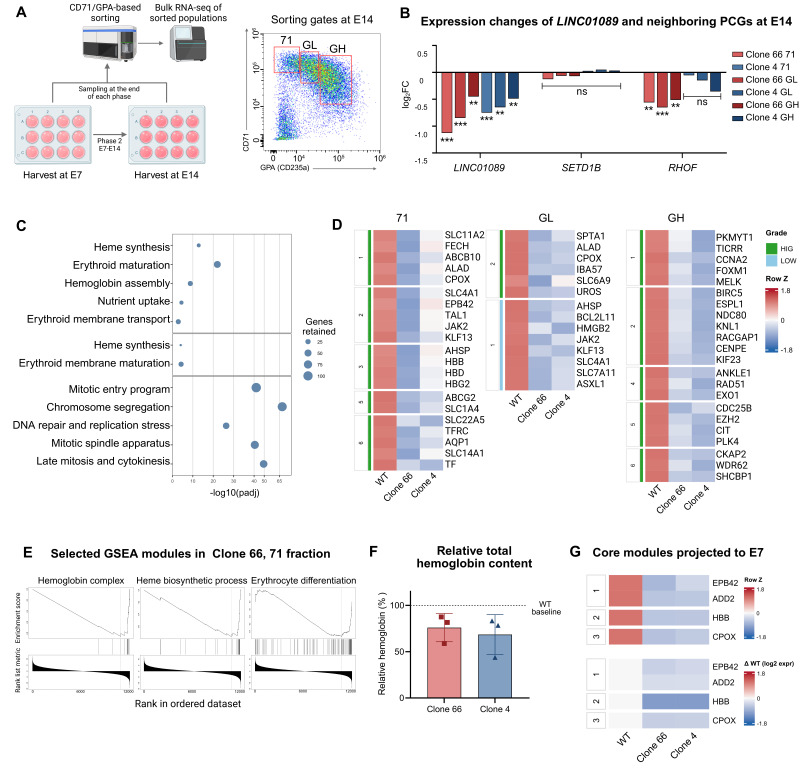
Bulk transcriptomics of stage-sorted erythroid subpopulations identifies a shared erythroid transcriptional defect core in *LINC01089*+/− clones. (**A**) Schematic of the bulk RNA sequencing (RNA-seq) workflow for stage-sorted erythroid subpopulations and representative sorting gates at E14. (**B**) Expression changes of *LINC01089* and its neighboring protein-coding genes (PCGs) across E14 erythroid fractions in *LINC01089*+/− clones relative to WT. *SETD1B* remained mostly unchanged, while *RHOF* showed modest and clone-limited changes. ** *p* < 0.01; *** *p* < 0.001; ns, not significant. (**C**) Shared modules identified by cross-clone enrichment-map (EM) clustering of downregulated terms. From top to bottom sections: CD71+GPA−/dim (71), CD71+GPAlow (GL), and CD71+GPAhigh (GH) fractions. The 71 and GL fractions were enriched for erythroid-centered modules, whereas the GH fraction additionally showed strong enrichment of mitotic entry, chromosome segregation, and late mitosis/cytokinesis modules. Dot size indicates the number of retained genes, and the x-axis indicates enrichment significance. The full cross-clone EM results are provided in Supplementary Dataset S8. (**D**) Heatmaps of representative genes from the modules shown in Figure 5C. The 71 and GL fractions showed coherent erythroid programs, including heme biosynthesis, hemoglobin-related, and membrane transport genes. In contrast, the GH fraction was dominated by mitotic and chromatin-associated regulators. The side color bar indicates between-clone gene-retention grade based on the 95% Jaccard overlap criterion: high (HIG; >0.25), moderate (MOD; 0.20–0.25), and low (LOW; <0.20). (**E**) Representative Gene Set Enrichment Analysis (GSEA) enrichment plots for the 71 fraction of Clone 66, illustrating coordinated downregulation of erythroid-related transcriptional programs. The complete GSEA results are provided in Supplementary Dataset S9. (**F**) Relative total hemoglobin content measured at E14 using a total hemoglobin assay kit based on absorbance at 400 nm (A400). Cell inputs were normalized to viable cell number, and the A400 signal was further normalized to the corresponding CD71+GPA+ erythroid fraction. Values are shown relative to the WT baseline. (**G**) Projection of the compact E14 defect-core gene panel onto the E7 dataset. Heatmaps are shown as row Z-scores and difference relative to WT (ΔWT; log2 expression difference relative to WT). A small set of genes, including *HBB*, was already significantly reduced at E7, indicating early signatures of the later E14 transcriptional defect. Complete summaries of erythroid-gene landscapes at E7 and E14 are provided in Supplementary Dataset S10.

To validate and refine the observations from scRNA-seq, we next performed bulk RNA sequencing (RNA-seq) on sorted erythroid subpopulations from six independent batches (three per time point) (Figure 5A). The erythroid compartment was gated into CD71+GPA−/dim (71), CD71+GPAlow (GL), and CD71+GPAhigh (GH) subpopulations at E14 and into a CD71+GPA+ (G) population at E7 (Figure S5A). Differential expression analysis results indicated broader and more dynamic transcriptomic changes at E14 than at E7 in both clones (Table S3; Supplementary Dataset S7).

As scRNA-seq may not reliably capture lncRNA expression changes, we examined *LINC01089* expression in the bulk datasets, which showed persistent partial suppression of *LINC01089* (Figure 5B and Figure S5B). To assess whether the deletion caused a local cis effect during erythroid culture, we examined the expression changes in its neighboring PCGs. The results showed much weaker and clone-limited changes in *SETD1B* and *RHOF* expression at E7 and E14 (Figure 5B and Figure S5B). These results argue against a broad local cis effect on neighboring PCGs during erythroid differentiation.

We next focused on the E14 dataset. Enrichment-map (EM) clustering of over-representation analysis (ORA)-enriched terms from the downregulated genes of each clone revealed clone-weighted differences alongside shared erythroid transcriptional changes. In the 71 and GL fractions, both clones showed enrichment for erythroid-related terms. Clone 4 was additionally dominated by strong chromosome segregation, mitotic nuclear division, and cell cycle signals across all subpopulations (−log_10_(adjusted *p*-value) > 40; Supplementary Dataset S11), whereas such signals only emerged in GH of Clone 66 (Figure S5C). In a stricter cross-clone EM strategy, a compact shared module core became evident (Figure 5C), consistent with the results highlighted by the single-cell analyses.

Gene-level heatmaps further revealed key transcriptional identities for these modules (Figure 5D and Figure S5D, Table S8). The 71 fraction showed representative heme-biosynthetic genes (*ALAD*, *CPOX*, *FECH*, and *ABCB10*), hemoglobin-related genes (*HBB*, *HBD*, *HBG2*, and *AHSP*), and membrane or transport genes (*TFRC*, *SLC11A2*, *SLC4A1*, and *EPB42*). In GL, this pattern remained evident but was more compact. By contrast, GH was enriched for mitotic and chromatin-associated genes, represented by *FOXM1*, *CCNA2*, *BIRC5*, and *NDC80*.

Representative heme-biosynthetic and globin-associated genes across E14 erythroid fractions showed coherent suppression of the heme–globin program in both *LINC01089*+/− clones, with *HBB* showing the most prominent reduction and *HBA1* showing weaker or less consistent changes (Figure S5E). Heme-biosynthetic genes, including *FECH*, *CPOX*, *UROS*, *UROD*, *ALAD*, and *SLC25A37*, were also reduced across multiple fractions. These results suggest impaired transcriptomic-level coordination between globin expression and heme-biosynthetic programs in *LINC01089*+/− cells during erythroid culture. Consistent with this imbalance, the *HBB*-to-*HBA1* expression ratio was reduced in most *LINC01089*+/− erythroid populations at E7 and E14 (Figure S5F). The higher *HBB*-to-*HBA1* ratio in the Clone 4 GH fraction may reflect selective retention of surviving CD71+GPAhigh cells under the stronger E14 viability defect.

In Gene Set Enrichment Analysis (GSEA), both clones repeatedly showed enrichment for erythroid differentiation, hemoglobin or oxygen transport, and heme biosynthetic or metabolic terms (Figure 5E and Figure S5G), supporting the same defect identified by the ORA results.

For a supportive non-transcriptomic readout, we measured absorbance at 400 nm (A400)-based total hemoglobin content at E14. The normalized total hemoglobin content showed a trend toward reduction in both *LINC01089*+/− clones compared with WT, without reaching statistical significance (Figure 5F; Supplementary Dataset S12), supporting that this impaired transcriptional coordination was accompanied by a trend toward reduced total hemoglobin accumulation.

Combining these results, a compact gene panel of the defective erythroid programs was constructed (Table S4). Projection of this panel onto the E7 dataset identified a small set of significant genes as early indicators (Figure 5G). A broader downregulation trend was also present at E7 without strict adjusted *p*-value filtering (Figure S5H). Notably, *HBB* was the most strongly reduced gene. Additional Human Phenotype Ontology (HPO) enrichment showed anemia- and abnormal hemoglobin-related terms in the 71 and GL fractions at E14, providing supportive disease relevance for the erythroid defects in our model (Table S5; Supplementary Dataset S13).

### 2.6. Early FAK Inhibition Partially Shifts Erythroid Transcriptional Readouts in Clone 66 LINC01089+/− Cells

**Figure 6:**
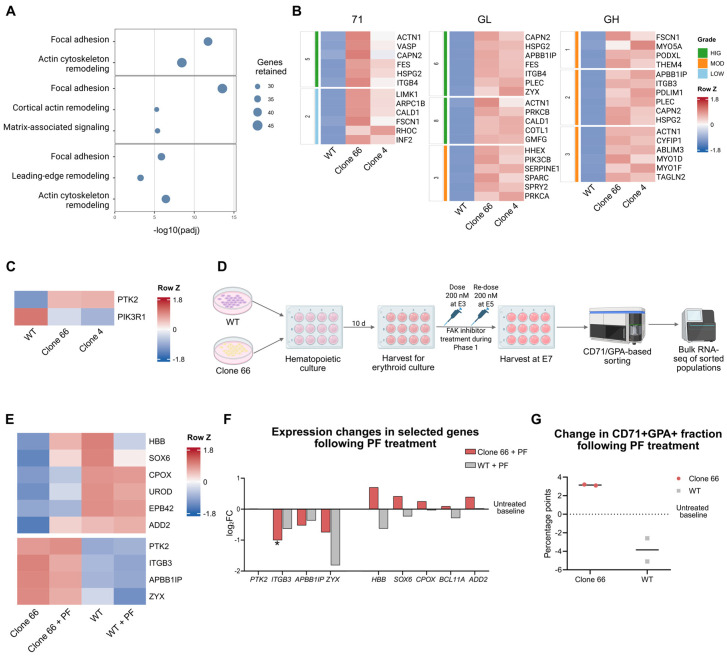
Shared focal adhesion signatures in *LINC01089*+/− cells and partial transcriptional and phenotypic responses to early focal adhesion kinase (FAK) inhibition. (**A**) Shared upregulated functional modules identified by cross-clone enrichment-map clustering of the E14 datasets. From top to bottom sections: 71, GL, and GH fractions. Focal adhesion modules were consistently detected across all erythroid fractions. (**B**) Heatmaps of representative genes from the shared modules shown in Figure 6A. The side color bar indicates between-clone gene-retention grade based on the 95% Jaccard overlap criterion: HIG (>0.25), MOD (0.20–0.25), and LOW (<0.20). (**C**) Projection of a selected FAK/phosphorylated FAK (pFAK)-proximal gene panel onto the E7 data. *PTK2* and *PIK3R1* were identified as representative early-responsive genes. (**D**) Schematic of the pilot FAK inhibitor (PF-573228; PF) perturbation experiment based on two independent biological comparisons. PF primarily suppresses FAK Tyr397 phosphorylation. WT and Clone 66 cells were treated during early erythroid culture, followed by CD71/GPA-based sorting and transcriptomic assessment at E7. (**E**) Heatmap of selected erythroid and FAK/pFAK-related genes in untreated and PF-treated WT and Clone 66 samples. Top, representative erythroid genes; bottom, representative focal adhesion/pFAK-proximal genes. *HBB*, *ADD2*, *EPB42*, and *CPOX* were included as early-responsive erythroid genes identified at E7. (**F**) Expression changes in selected genes following PF treatment. Bars indicate log2 fold changes relative to untreated controls. In Clone 66, *HBB* and other erythroid genes (*CPOX* and *ADD2* as early defect indicators) showed partial transcriptional shifts toward the WT control state, whereas PF-treated WT samples showed the opposite trend. Most FAK/pFAK-related genes were reduced in all PF-treated samples, with *ITGB3* showing the strongest reduction. PF did not significantly alter *PTK2* expression, consistent with inhibition of Tyr397 phosphorylation rather than *PTK2* messenger RNA (mRNA) expression. * *p* < 0.05. (**G**) Change in the CD71+GPA+ fraction following PF treatment. Values indicate percentage-point changes relative to the matched untreated baseline. Each point represents an independent pilot treatment comparison. Cell viability and count data showed minor changes and are summarized in Supplementary Dataset S14.

As analyses of the downregulated genes mainly defined downstream erythroid defects, we analyzed whether an upregulated signaling axis might contribute to these abnormalities. Cross-clone EM clustering of the E14 upregulated datasets identified consistent focal adhesion signals in all erythroid fractions (Figure 6A).

In 71, genes such as *ACTN1*, *VASP*, *CAPN2*, and *ITGB4* suggested a focal adhesion state toward receptor input and upstream activities (Figure 6B and Figure S6A, Table S9). In GL, *CAPN2*, *APBB1IP*, *ITGB4*, *PLEC*, and *ZYX* pointed more to adhesion signal processing. In GH, genes including *ITGB3*, *PDLIM1*, *PLEC*, *CYFIP1*, and *TAGLN2* indicated peripheral and downstream remodeling readouts. Among these genes, a pFAK-proximal subset of genes emerged, represented by *ITGB4* (71, GL), *ITGB3* (GH), *CAPN2* (71, GL, GH), and *APBB1IP* (GL, GH). Together with prior literature reporting that *LINC01089* can suppress FAK phosphorylation [21], this pattern provided a rationale for investigating the FAK/pFAK signaling axis.

A FAK/pFAK gene panel (Table S6) based on the pFAK-proximal genes was constructed and projected to the E14 and E7 datasets (Figure 6C and Figure S6B). The E7 results yielded only *PTK2* itself and *PIK3R1* as significant genes. However, it also suggested that the FAK-related genes had already started shifting (Figure S6C).

To explore whether this axis was functionally relevant, we designed a pilot pFAK perturbation experiment (n = 2) with the FAK inhibitor PF-573228 (PF) during early erythroid culture (Figure 6D). In Clone 66, *HBB* expression showed a partial increase after treatment, with other erythroid genes displaying weaker responses in the same direction (Figure 6F and Figure S6D). By contrast, the treated WT samples showed reduced *HBB* expression. Among the pFAK-related genes, several key genes were reduced after PF treatment, with *ITGB3* showing the clearest change. Flow-cytometry analysis also showed modest changes in opposite directions between Clone 66 and WT in the CD71+GPA+ fraction after PF treatment, with only minor changes in total cell numbers and viability (Figure 6G and Figure S6E; Supplementary Dataset S14).

## 3. Discussion

In this study, we examined the erythroid phenotypes of *LINC01089*+/− perturbation in a human hESC-derived differentiation model. Reduced *LINC01089* expression produced coherent erythroid defects, a conclusion supported by consistent evidence across multiple data layers in two independent clones. Our findings point to impaired establishment of coordinated heme–globin transcriptional programs that are essential for erythroid differentiation [23,24]. In Clone 66, the FAK/pFAK intervention produced partial shifts of erythroid gene expression and CD71+GPA+ fraction toward untreated-WT-like states, supporting FAK-related signaling as a potential contributing axis to the defective phenotype. Our results provide, to our knowledge, the first evidence linking *LINC01089* to human erythroid biology and establish an hESC-derived platform for future nomination and mechanistic studies of lncRNA candidates in erythroid differentiation and other long-range differentiation models.

Although hESC erythroid culture does not fully recapitulate physiological erythropoiesis in vivo, it permits a stable genetic background for perturbation and multi-layer phenotypic analysis. Two well-established complementary erythroid culture models were adopted. The AGM-S3 co-culture model ensured a higher screen input capacity, while the STEMdiff erythroid kit provided a more chemically defined and erythroid-biased model for validation [25]. However, because the present study ended at the E14 culture endpoint, maturation-related interpretations should be understood within the erythroblast-stage window captured at E14, without implying completed terminal maturation or enucleation.

The CRISPR pgRNA strategy produced a mild reduction in *LINC01089* expression in both clones at the hESC stage, and the reduction became stronger during erythroid culture. This is consistent with previous studies, as heterozygous deletion of a lncRNA genomic segment may not necessarily produce a linear 50% reduction in RNA expression [26], and the expression change can be cell-context dependent for the same deletion design [27].

We highlight the consistency of the erythroid defect across phenotypic and transcriptomic layers, while maintaining clone-specific resolution. scRNA-seq indicated distinct erythroid subcluster-state variation in the two clones, with a coherent pattern of a weakened hemoglobin maturation module toward later subclusters (Figure S4A). Bulk transcriptomic profiling strengthened this interpretation, consolidating the defect around genes related to hemoglobin, heme biosynthesis, and membrane maturation. ORA and GSEA results converged on the same core abnormalities, suggesting both strong focal signals and a broader transcriptomic shift.

These defects were also accompanied by the downregulation of nuclear division and chromosome segregation transcriptional programs. Erythroid differentiation requires coordinated progression through the final mitotic divisions, followed by orderly cell-cycle exit, chromatin condensation, and nuclear remodeling [28,29,30]. This broad suppression pattern in GH suggests a disturbed progression toward maturation, consistent with insufficient maintenance and early tapering of these programs.

The downregulation of these transcriptional programs showed clone-weighted differences at E14, which were detected across all subpopulations of Clone 4, but mainly in the GH fraction of Clone 66. This suggests that Clone 4 may be more vulnerable to cell-cycle and chromosome-segregation program dysregulation during erythroid culture, consistent with its stronger reduction in cell expansion and viability at E14. We therefore used Clone 66 for deeper scRNA analysis and subsequent PF-573228 perturbation experiments because of its milder phenotype. The colony assay results also showed clone-weighted trends in BFU-E-to-CFU-E ratio and erythroid colony outputs. Nevertheless, these clone-weighted differences did not alter the shared erythroid transcriptional defect core identified in both clones.

Interestingly, our results showed an early and progressive reduction in *HBB* expression, which emerged before the broader defect pattern was fully established (Figure 5G and Figure S5E,F,H). Prior studies in β-thalassemia have reported that reduced *HBB* expression leads to α/β-globin imbalance and that excess unpaired α-globin chains can promote oxidative, membrane, and apoptotic stress [31,32,33]. Human erythroid *HBB*-loss models have shown impaired hemoglobinization, increased apoptosis, and slower proliferative or cell-cycle progression [34,35]. These findings are consistent with our observation of impaired heme–globin programs and raise the possibility that the downregulation of chromosome segregation and mitotic programs observed in the E14 GH fraction may occur as a consequence of impaired heme–globin program establishment, a possibility worth further investigation. Importantly, the A400 assay provided only a non-β-globin-specific readout of total hemoglobin accumulation. Thus, *HBB* remained a transcriptional anchor in our study. Similarly, the *HBB*-to-*HBA1* ratio was interpreted as a transcriptomic-level measure of globin balance rather than a clinical red blood cell index.

Another notable observation was the recurrent focal adhesion signal. Integrin-FAK signaling has been implicated in stress erythropoiesis and in microenvironmental support for erythroid development [36,37,38,39]. Consistent with a recent report that *LINC01089* directly inhibits FAK phosphorylation [21], our E14 analysis highlighted a pFAK-proximal gene set represented by *ITGB3*, *ITGB4*, *APBB1IP*, and *CAPN2*. Here, *ITGB3* and *ITGB4* may represent candidate integrin inputs linked to pFAK activation [40,41], *APBB1IP* may support integrin–talin coupling [42], and *CAPN2* may indicate FAK proteolysis or remodeling [43]. The expression pattern of this gene set suggests a dynamic reconfiguration of the FAK/pFAK signaling neighborhood at E14 during erythroid differentiation (Figure S6B). Together, the opposite-direction responses between PF-treated Clone 66 and WT samples in both transcriptional and flow-cytometry readouts (Figure 6F,G) raise the possibility that excessive or mis-tuned FAK activity may contribute to impaired erythroid progression, implying a possible fine-tuning role of *LINC01089* in this axis.

Collectively, our data support a role for *LINC01089* in maintaining the coordinated erythroid expression programs associated with erythroid maturation. In this sense, *LINC01089* may be viewed alongside an established class of erythroid lncRNAs functionally coupled to key erythroid developmental components. Such lncRNAs include *GATA2-AS1*, *UCA1*, *lincRNA-EPS*, and *Bloodlinc*, which have been reported to modulate TF occupancy and chromatin state, stabilize heme-synthesis machinery, set apoptotic thresholds, or coordinate terminal erythroid programs and output [9,10,11,44]. In this context, our study links *LINC01089* to an *HBB*-centered erythroid transcriptional defect pattern, with possible involvement of the FAK/pFAK axis.

In addition, we cautiously interpret the HPO enrichment results (Table S5). FAK is a well-recognized drug target whose activity can be modulated by small-molecule inhibitors [45]. It has been explored extensively in cancer research, including in a combination therapy approved by the U.S. Food and Drug Administration (FDA) [46]. Together, these findings suggest that *LINC01089* may be worth further investigation in erythroid disorders characterized by impaired erythroid output, reduced *HBB* expression, or abnormal maturation, with possible involvement of the FAK/pFAK axis.

Several limitations should be acknowledged. First, homozygous *LINC01089* deletion clones were not obtained. This may be locus- and cell-line-dependent for lncRNA locus editing [47], and homozygous clones may not be obtained after screening hundreds of clones [27]. Second, the current data cannot completely exclude potential off-target effects introduced during CRISPR editing. Clone-to-clone hESC background variation may also arise during clonal line establishment, consistent with reported differences in hematopoietic differentiation potential among hESC lines [48]. These clone-specific factors could potentially contribute to clone-specific severity, but are unlikely, on their own, to fully account for the shared erythroid defects observed in both clones. Third, we did not establish transcript-isoform-specific rescue or optimized orthogonal knockdown. Effective knockdown of *LINC01089* was not achieved with three small interfering RNA (siRNA) designs targeting multiple *LINC01089* transcripts during erythroid culture. Therefore, we used a CRISPR-based strategy to perturb the *LINC01089*-transcribed genomic region. Future antisense oligonucleotide (ASO)-based or isoform-specific rescue experiments will be needed to define the erythroid mechanism of *LINC01089*.

In conclusion, this study identifies *LINC01089* as a novel lncRNA linked to coordinated erythroid maturation-associated programs, with an *HBB*-centered transcriptional defect and possible involvement of the FAK/pFAK axis. Our findings provide a foundation for future mechanistic studies and for exploring the role of *LINC01089* in erythroid diseases.

## 4. Materials and Methods

### 4.1. Cell Lines and Maintenance

H1 hESCs were provided by Prof. Tao Cheng (Institute of Hematology and Blood Diseases Hospital, CAMS & PUMC). A doxycycline-inducible Tet-On CRISPR/Cas9 system was obtained from Addgene (Watertown, MA, USA; plasmid no. 37500) and introduced into H1 cells according to the manufacturer’s instructions.

H1 cells were maintained on Matrigel (Corning, Corning, NY, USA; 354277)-coated plates in NcTarget™ hPSC Medium (Shownin Biotechnologies Co., Ltd., Hefei, China), with daily medium changes. Cells were passaged at approximately 80% confluence using ReLeSR (STEMCELL Technologies Canada Inc., Vancouver, BC, Canada). Routine mycoplasma testing was performed every 2 weeks in undifferentiated hESC cultures using a rapid mycoplasma detection kit (BRK0043, ABclonal, Wuhan, China).

### 4.2. Generation of LINC01089-Edited Clones

Two sgRNAs flanking the *LINC01089* target region were designed using the CRISPOR web tool (https://crispor.org; accessed on 7 May 2026) with the *Streptococcus pyogenes* Cas9 (SpCas9) NGG protospacer-adjacent motif (PAM) setting based on balanced target-region coverage and predicted off-target profiles. H1 cells were dissociated into single cells with Accutase (STEMCELL Technologies Canada Inc., Vancouver, BC, Canada) and nucleofected with two sgRNA-Cas9 plasmids (5 μg each) using the Lonza P3 Primary Cell 4D-Nucleofector Kit (Lonza, Basel, Switzerland) with manufacturer-provided 4D-Nucleofector program CB-150. Cells were then plated for recovery and expanded for single-clone isolation and validation. Genomic DNA was extracted from expanded clones for PCR amplification. Deletion-junction and retained WT-allele bands were analyzed by agarose gel electrophoresis. PCR products spanning the deletion junctions in Clone 66 and Clone 4 were subjected to Sanger sequencing. hESC-stage *LINC01089* expression was assessed by reverse transcription quantitative polymerase chain reaction (RT-qPCR). sgRNA sequences and primers used for genotyping, junction validation, and RT-qPCR are listed in Table S7. The CRISPOR off-target prediction summary is provided in Supplementary Dataset S3.

### 4.3. Erythroid Culture Models

In both models, mycoplasma testing was performed at each harvest point and no antibiotics were added to ensure authentic phenotypic results.

#### 4.3.1. AGM-S3 Co-Culture Model

As described previously [15,16], hESC aggregates were seeded onto irradiated AGM-S3 feeder cells (13.5 Gy). After 3 days of maintenance culture, hematopoietic differentiation was induced by medium change. To generate erythroid cells from the co-culture model, day-12 cells were collected and seeded at 1 × 10^5^ cells/mL in 12-well plates for a two-phase liquid erythroid differentiation culture, as described in the Supplementary Material.

#### 4.3.2. STEMdiff Erythroid Differentiation Model

Cultures were established using the STEMdiff™ Erythroid Kit (STEMCELL Technologies Canada Inc., Vancouver, BC, Canada) according to the manufacturer’s instructions. For optimized hematopoietic stem and progenitor cell (HSPC) yield, fewer cell aggregates were seeded at the initial stage. The optional phase 3 maturation and enucleation step was not included in the present study.

### 4.4. Pooled pgRNA Screening

The pgRNA library was obtained from Addgene (Watertown, MA, USA; Pooled Library #89640), targeting 671 lncRNAs and 20 PCGs with 12,452 pgRNA pairs. Library construction was performed according to a published protocol [49]. Lentiviral packaging was outsourced to Jiangsu Puzhu Biopharmaceutical Technology Co., Ltd. (Nanjing, China).

The screening system involved optimization-driven compromises to balance input size and library representation during long-range culture, a challenge also reported in a hESC-derived organoid screening of comparable scale [50]. Detailed screening design and processing steps are described in the Supplementary Material.

### 4.5. Flow Cytometry and Cell Sorting

For non-erythroid analysis, cells were washed and resuspended in phosphate-buffered saline (PBS) containing 2% fetal bovine serum (FBS) and 1 μM ethylenediaminetetraacetic acid (EDTA), hereafter referred to as FC-PBS. Samples were stained on ice for 30 min with anti-CD34 (BD Biosciences, Franklin Lakes, NJ, USA; clone 581) and anti-CD43 (BD Biosciences, Franklin Lakes, NJ, USA; clone 1G10) antibodies, washed, and resuspended in cold FC-PBS. For erythroid analysis and sorting, EDTA was omitted from the staining buffer to reduce additional stress during cell sorting. Anti-CD71 (BD Biosciences, Franklin Lakes, NJ, USA; clone M-A712) and anti-CD235a (BioLegend, San Diego, CA, USA; clone HI264) antibodies were used for erythroid analyses and sorting. Flow cytometry was performed on a DxFLEX flow cytometer (Beckman Coulter Life Sciences, Indianapolis, IN, USA), and cell sorting was carried out using a CytoFLEX SRT sorter (Beckman Coulter Life Sciences, Indianapolis, IN, USA) according to the indicated gating strategy (Figure 5A and Figure S5A). Data were analyzed using FlowJo software (v10.8.1).

### 4.6. Cell Counting and Viability Assessment

Collected cells were resuspended in appropriate volumes (typically 0.5 or 1 mL). For cell counting and viability assessment, 10 μL of cell suspension was mixed 1:1 with acridine orange/propidium iodide (AO/PI) staining solution (Countstar/Alit Biotech (Shanghai) Co., Ltd., Shanghai, China; lot no. J24023) and loaded onto the Countstar Rigel S2 Fluorescence Cell Analyzer (Countstar/Alit Biotech (Shanghai) Co., Ltd., Shanghai, China) according to the manufacturer’s instructions.

### 4.7. Hematopoietic Colony Assay

Colony assays were performed using MethoCult™ H4636 (STEMCELL Technologies Canada Inc., Vancouver, BC, Canada). For each sample, 2 × 10^4^ day-10 cells derived from the STEMdiff erythroid culture model were plated according to the manufacturer’s instructions. BFU-E, CFU-E, and granulocyte-macrophage (GM) colonies were scored after 14 days of culture.

### 4.8. Total Hemoglobin Assay

Total hemoglobin at E14 was estimated using a Hemoglobin (Hb) Content Assay Kit (Solarbio, Beijing, China; BC5585) according to the manufacturer’s instructions. Cells were analyzed directly from total erythroid cultures without sorting to avoid additional handling stress. For each batch, assay input was normalized by loading an equal number of viable cells per sample. Absorbance at 400 nm was measured, and blank-corrected values were further normalized to the corresponding CD71+GPA+ erythroid fraction measured by flow cytometry. Values were presented relative to the WT baseline as an estimated total hemoglobin-content readout.

### 4.9. Single-Cell RNA Sequencing and Analysis

Total cells collected at E7 and E14 were subjected to single-cell RNA sequencing. Approximately 25,000 cells from each sample were loaded onto the BGI single-cell platform (BGI Genomics, Shenzhen, Guangdong, China). Raw data were processed using fastp v0.23.4 and DNBC4tools v2.1.3 to generate gene expression matrices. Doublets and high-mitochondrial-content cells were filtered. Downstream analyses were performed using the Majorbio Cloud platform (Shanghai Majorbio Bio-pharm Technology Co., Ltd., Shanghai, China; www.majorbio.com; accessed on 7 May 2026) with Seurat 5.0.1 for dimensionality reduction, clustering, subclustering, and module scoring, and Monocle 2.22.0 for pseudotime analysis.

### 4.10. Bulk RNA Sequencing and Downstream Bioinformatic Analyses

Sorted subpopulations were immediately lysed in TRIzol (Thermo Fisher Scientific, Waltham, MA, USA) upon collection and submitted to GENEWIZ (Azenta Life Sciences, Suzhou, China) for RNA sequencing. Gene-level counts were quantified using HTSeq (v0.6.1). Raw count matrices were processed in DEBrowser (v1.38.0) for normalization and differential expression analysis. Rank-based pathway analysis was performed in WebGestalt (https://www.webgestalt.org; accessed on 7 May 2026) using full ranked gene lists, and threshold-based ORA was performed using g:Profiler through a version-pinned gprofiler2 (v0.2.4) workflow (https://biit.cs.ut.ee/gprofiler; archive endpoint e113_eg59_p19; accessed on 7 May 2026). Enrichment results were further organized in Cytoscape (v3.10.4) with EnrichmentMap (v3.5.0) to evaluate both clone-specific and cross-clone convergent patterns. Gene-level heatmaps were generated in R (version 4.5.2) using custom plotting scripts.

### 4.11. PF-573228 Treatment

PF-573228 (MedChemExpress, Monmouth Junction, NJ, USA) was dissolved in cell-culture-grade dimethyl sulfoxide (DMSO; MedChemExpress, Monmouth Junction, NJ, USA) and aliquoted for storage at −80 °C. The stock solution was diluted and administered on days 3 and 5 of phase 1 erythroid differentiation (E3 and E5) in the STEMdiff culture model at a final concentration of 200 nM. Only freshly thawed PF aliquots were used for each administration. Samples were collected at E7 for cell sorting and bulk RNA-seq.

### 4.12. Primers and Oligonucleotides

Primer and oligonucleotide sequences used in this study are provided in Table S7.

### 4.13. Statistical Analysis

Statistical analyses were performed using GraphPad Prism (v8.0.1). Data are presented as mean ± standard deviation. Individual replicate values are shown as data points for n ≤ 3 experiments, whereas individual points were omitted for larger replicate sets to preserve figure readability. Comparisons were performed primarily using one-way or two-way analysis of variance (ANOVA), followed by appropriate post hoc multiple-comparison tests where applicable. * *p* < 0.05, ** *p* < 0.01, *** *p* < 0.001, and **** *p* < 0.0001 were considered statistically significant.

## Data Availability

The raw sequencing datasets generated in this study, including the pgRNA screen sequencing data and transcriptomic sequencing data, have been deposited in the NCBI Sequence Read Archive (SRA) under BioProject accession number PRJNA1444776. The data will be made publicly available upon publication. Processed data are available in the article and its Supplementary Materials (Datasets S1–S14). Due to file-size limitations, the full single-cell expression matrix is not included in the Supplementary Materials and is currently available from the corresponding author upon request.

## Supplementary Materials

The following supporting information can be downloaded at: https://www.mdpi.com/article/10.3390/ijms27104394/s1.
