# Supplementary material for: Reduced LINC01089 Expression Impairs Coordinated Heme–Globin Transcriptional Programs in Human Erythroid Cells
Source: Int J Mol Sci. 2026 May 14;27(10):4394. doi: 10.3390/ijms27104394 (PMC13206774; doi:10.3390/ijms27104394)
Supplement: Supplementary file 1 [file ijms-27-04394-s001.zip › Supplementary Figures.pdf]

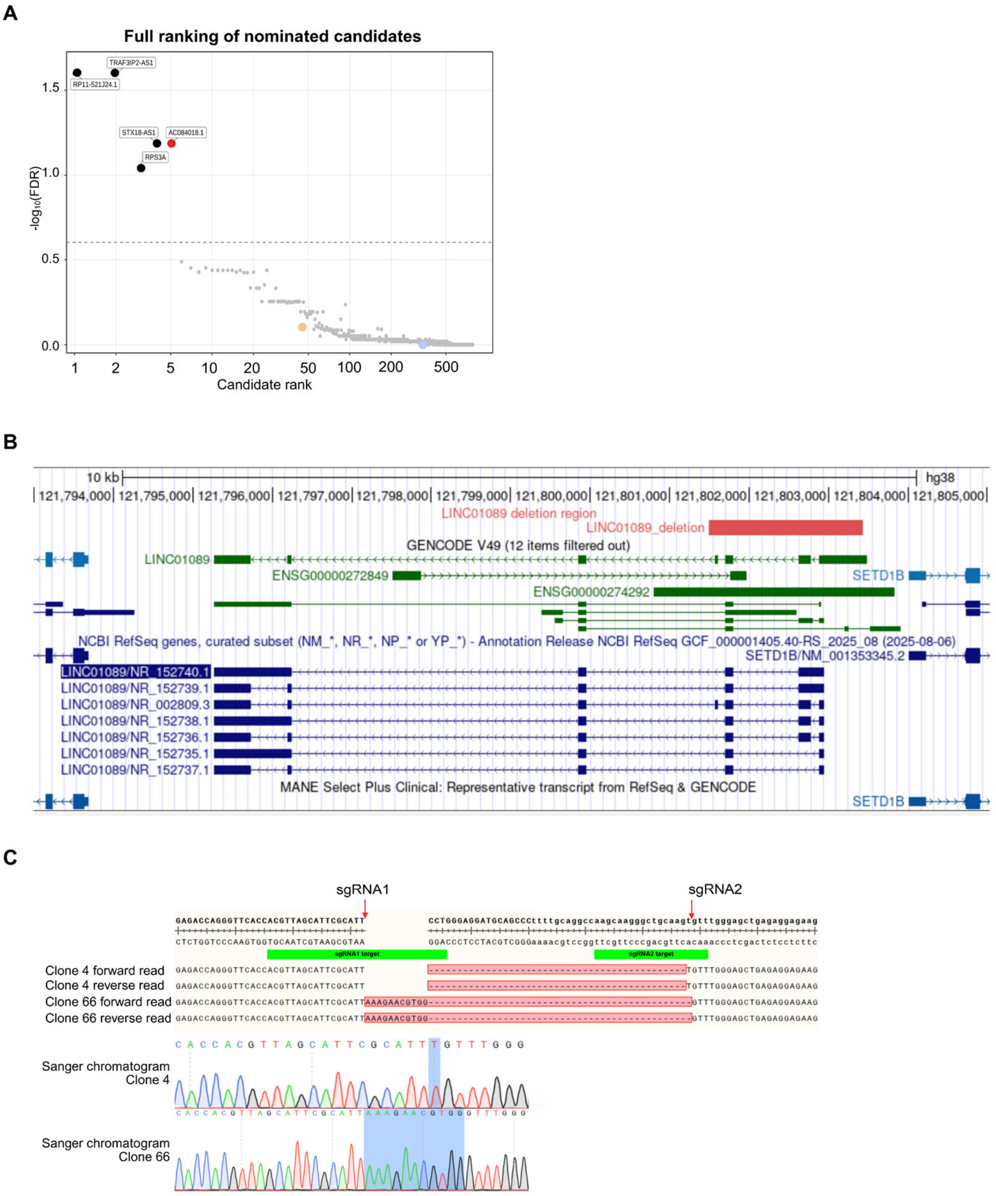

Figure S1. Full Model-based Analysis of Genome-wide CRISPR-Cas9 Knockout (MAGeCK) screen ranking and validation of the CRISPR/Cas9-mediated deletion junctions. (A) Full candidate ranking from the pooled paired-guide RNA (pgRNA) screen. Candidate targets are displayed according to screen rank and corresponding −log10(FDR) values, with FDR defined as the false discovery rate. *LINC01089* (AC084018.1) is highlighted in red. The dashed line denotes the nominal significance cutoff. The light orange dot represents non-targeting controls, and the light blue dot represents *AAVS1*-targeting pgRNAs. (B) Genome browser view of the *LINC01089*

locus showing annotated transcript models from GENCODE v49 and NCBI RefSeq, together with the CRISPR/Cas9 deletion interval used for clone generation. The red bar marks the *LINC01089*-transcribed genomic segment deleted for clone generation, which overlaps major annotated *LINC01089* transcript variants. (C) Sanger sequencing validation of the deletion junctions in Clone 4 and Clone 66. Forward reads and reverse-complemented reverse reads were aligned to the wild-type (WT) genomic sequence spanning the two single-guide RNA (sgRNA) cut sites, indicated by red arrows. Representative chromatograms across the repair junction are shown below. Blue shading marks the inserted bases at the junction, including a single T insertion in Clone 4 and an 11-bp junctional insertion in Clone 66.

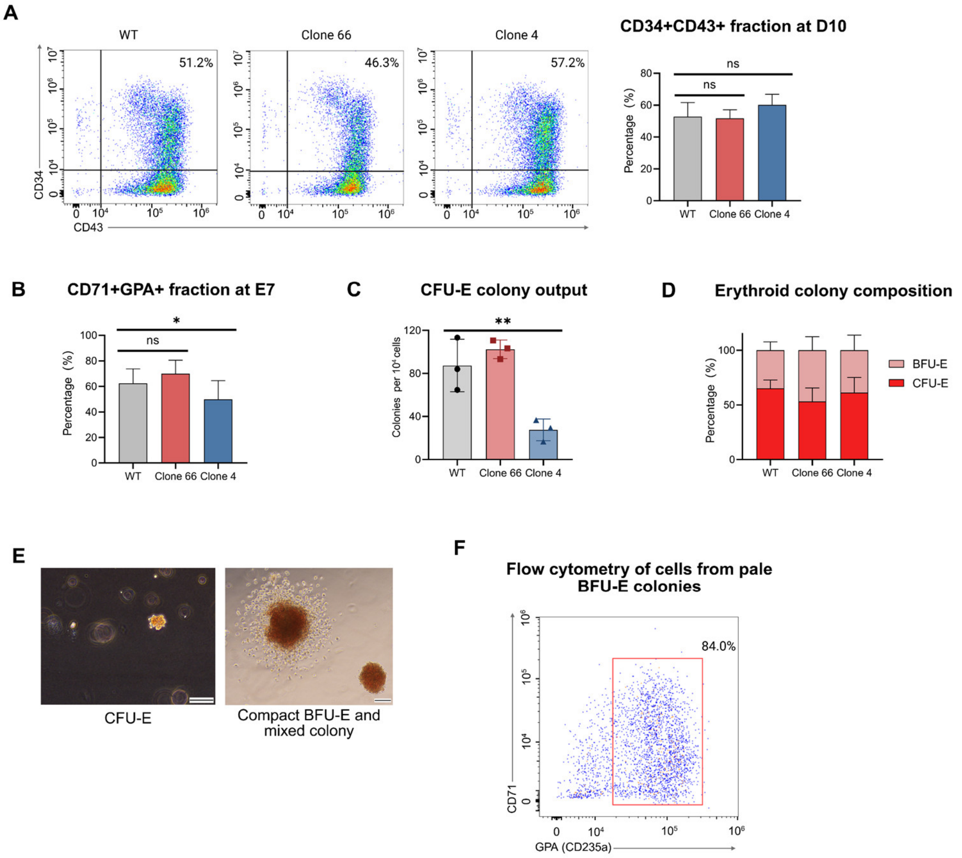

Figure S2. Additional phenotypic characterization during hematopoietic and erythroid culture, and colony analysis. (A) Representative flow-cytometry plots and quantification of the CD34+CD43+ fraction at D10. No significant differences were observed in this fraction (n = 8). (B) Quantification of the CD71+glycophorin A (GPA)+ fraction at E7 (n = 12). Changes at this stage were modest and clone-dependent. (C) Colony-forming unit-erythroid (CFU-E) colony output in WT, Clone 66, and Clone 4 samples, shown as colonies per 10^4 cells (n = 3). (D) Distribution of burst-forming unit-erythroid (BFU-E) and CFU-E colonies within the erythroid colony compartment, shown as percentages of total erythroid colonies (n = 3). (E) Representative bright-field images of colony types identified in the colony-forming assay from WT samples, including CFU-E and a compact BFU-E adjacent to a mixed colony. Scale bars, 100 μm. (F) Representative flow-cytometry plot of manually picked pale colonies from *LINC01089*+/− samples, showing that most cells remained within the erythroid GPA+ gate.

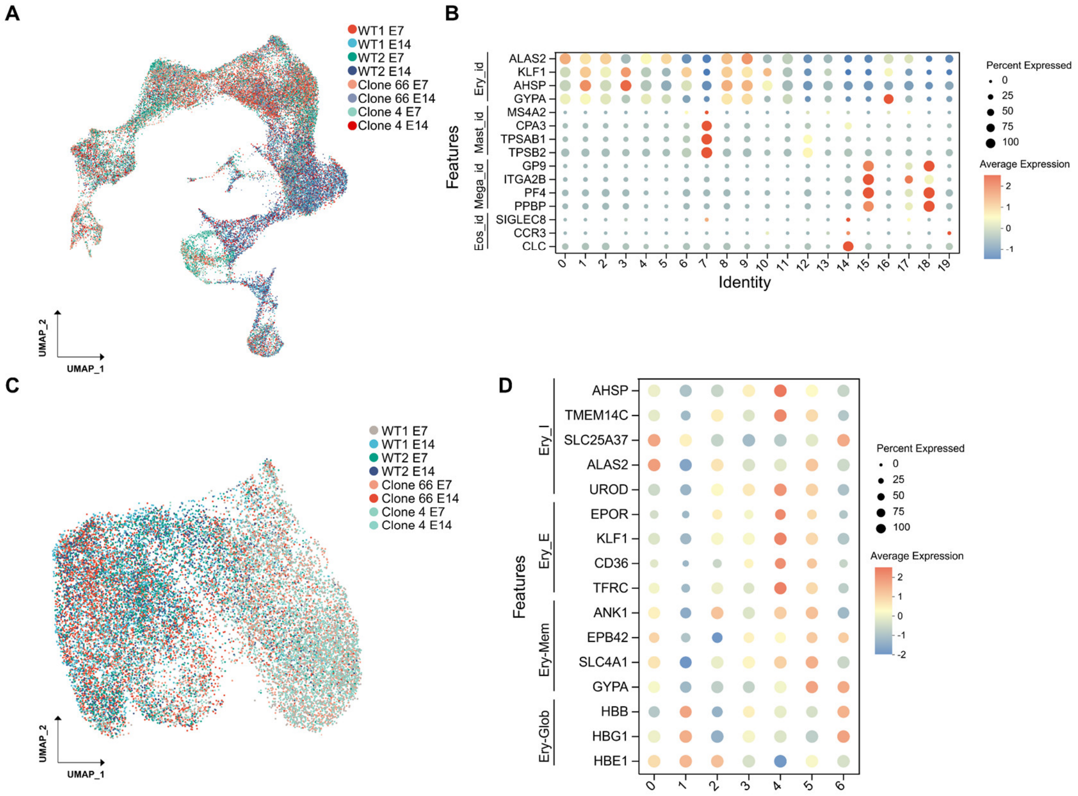

Figure S3. Single-cell annotation and sample-level distributions in the global and erythroid embeddings. (A) Uniform manifold approximation and projection (UMAP) visualization of the integrated single-cell RNA sequencing (scRNA-seq) dataset colored by sample and time point. (B) Dot plot of representative marker genes used for global cluster annotation. Clusters 3, 5, 10, and 16 were excluded from the erythroid compartment because they showed mixed identity, stress-associated features, immediate stress-response features, or residual erythroid-like signals despite partial erythroid marker expression. (C) UMAP visualization of the re-clustered erythroid compartment colored by sample and time point. (D) Dot plot of representative marker genes used to annotate erythroid subclusters. Abbreviations: Ery_I, intermediate erythroid program; Ery_E, early erythroid program; Ery-Mem, erythroid membrane maturation program; Ery-Glob, erythroid globin program.

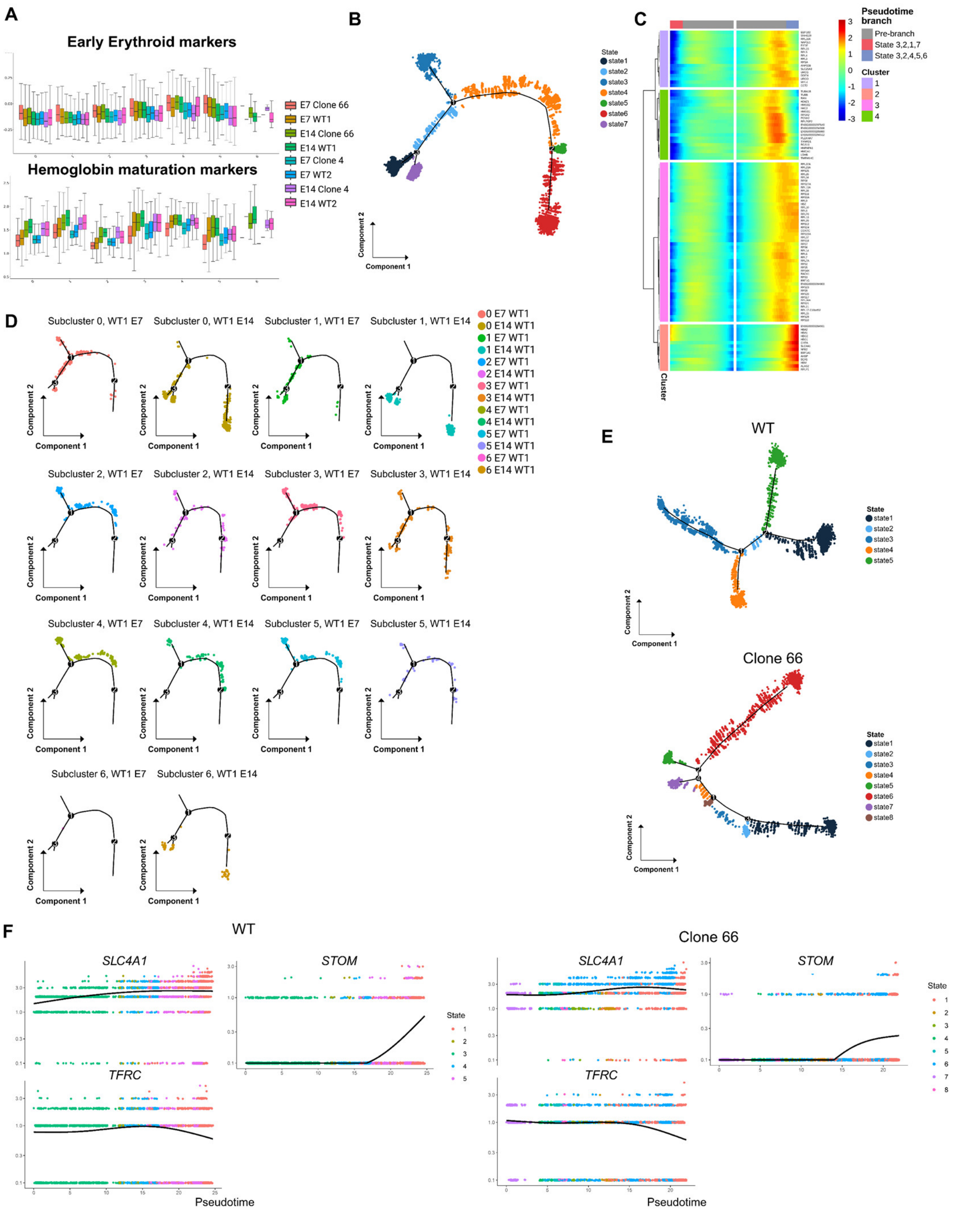

Figure S4. Single-cell program scoring and pseudotime analyses of erythroid maturation-associated programs. (A) Module scoring of representative early erythroid and hemoglobin maturation gene sets across erythroid subclusters at E7 and E14 in all samples. Hemoglobin maturation scores showed a consistently reduced pattern in mid-to-late subclusters (subclusters 0, 1, and 6) in both clones. (B) Monocle pseudotime trajectory reconstructed from erythroid cells, showing the major branch structure and state assignments. (C) Branch Expression Analysis Modeling (BEAM) heatmap at node 1 showing branch-dependent gene-expression patterns along the

erythroid trajectory. Genes were grouped into four expression clusters that broadly represent early biosynthetic/mitochondrial-support activity (cluster 1), structural-remodeling and early maturation-support features (cluster 4), ribosome/translation-dominant biosynthetic activity (cluster 3), and erythroid maturation associated with hemoglobinization, membrane maturation, and heme biosynthesis (cluster 2). (D) Projection of individual WT1 erythroid subclusters at E7 and E14 onto the pseudotime trajectory, showing the relative positions of subclusters along the maturation continuum. Late-state erythroid subclusters were mapped onto both erythroid pseudotime branches, supporting that the two branches reflect WT erythroid-state variation rather than a clone-specific editing artifact. (E) Pseudotime reconstructions of WT1 and Clone 66 erythroid cells without batch correction, colored by Monocle state. State numbers were defined independently within each trajectory and are not intended to indicate direct connections between the two samples. (F) Representative pseudotime expression dynamics of *SLC4A1*, *STOM*, and *TFRC* in WT and Clone 66 erythroid cells, illustrating differences in membrane-maturation and early erythroid marker dynamics along the trajectory.

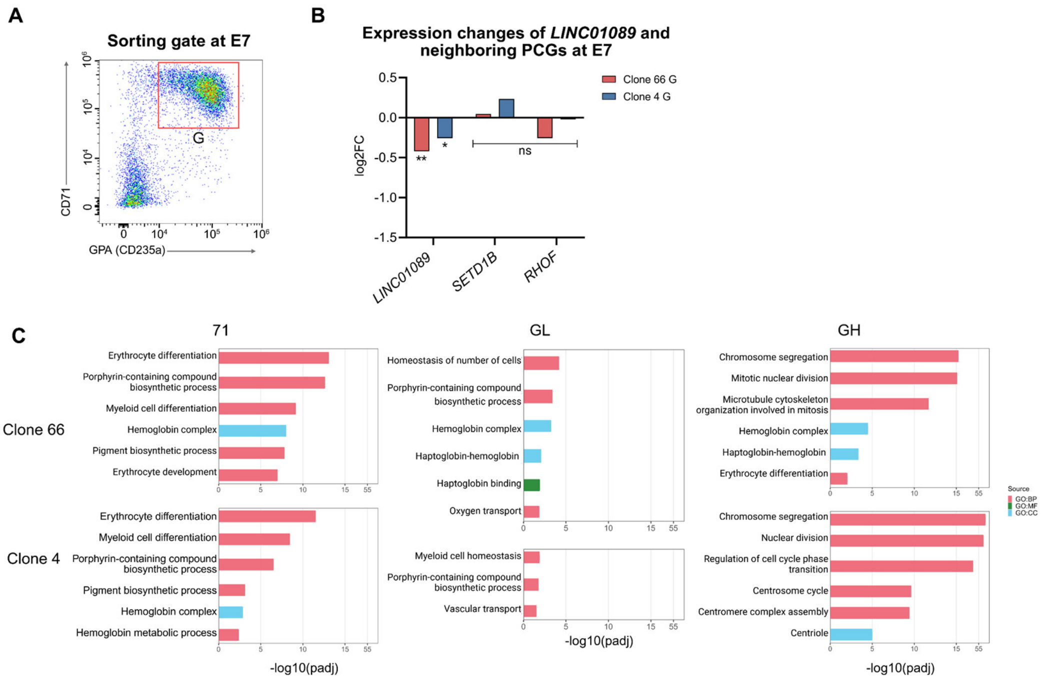
A
Sorting gate at E7
CD71
GPA (CD235a)
G
B
Expression changes of LINC01089 and neighboring PCGs at E7
Clone 66 G
Clone 4 G
log2FC
ns
LINC01089
SETD1B
RHOF
C
71
GL
GH
Clone 66
Clone 4
Erythrocyte differentiation
Porphyrin-containing compound biosynthetic process
Myeloid cell differentiation
Hemoglobin complex
Pigment biosynthetic process
Erythrocyte development
Hemoglobin metabolic process
Homeostasis of number of cells
Haptoglobin-hemoglobin
Haptoglobin binding
Oxygen transport
Myeloid cell homeostasis
Vascular transport
Chromosome segregation
Mitotic nuclear division
Microtubule cytoskeleton organization involved in mitosis
Nuclear division
Regulation of cell cycle phase transition
Centrosome cycle
Centromere complex assembly
Centriole
-log10(padj)
Source
GO:BP
GO:MF
GO:CC

D

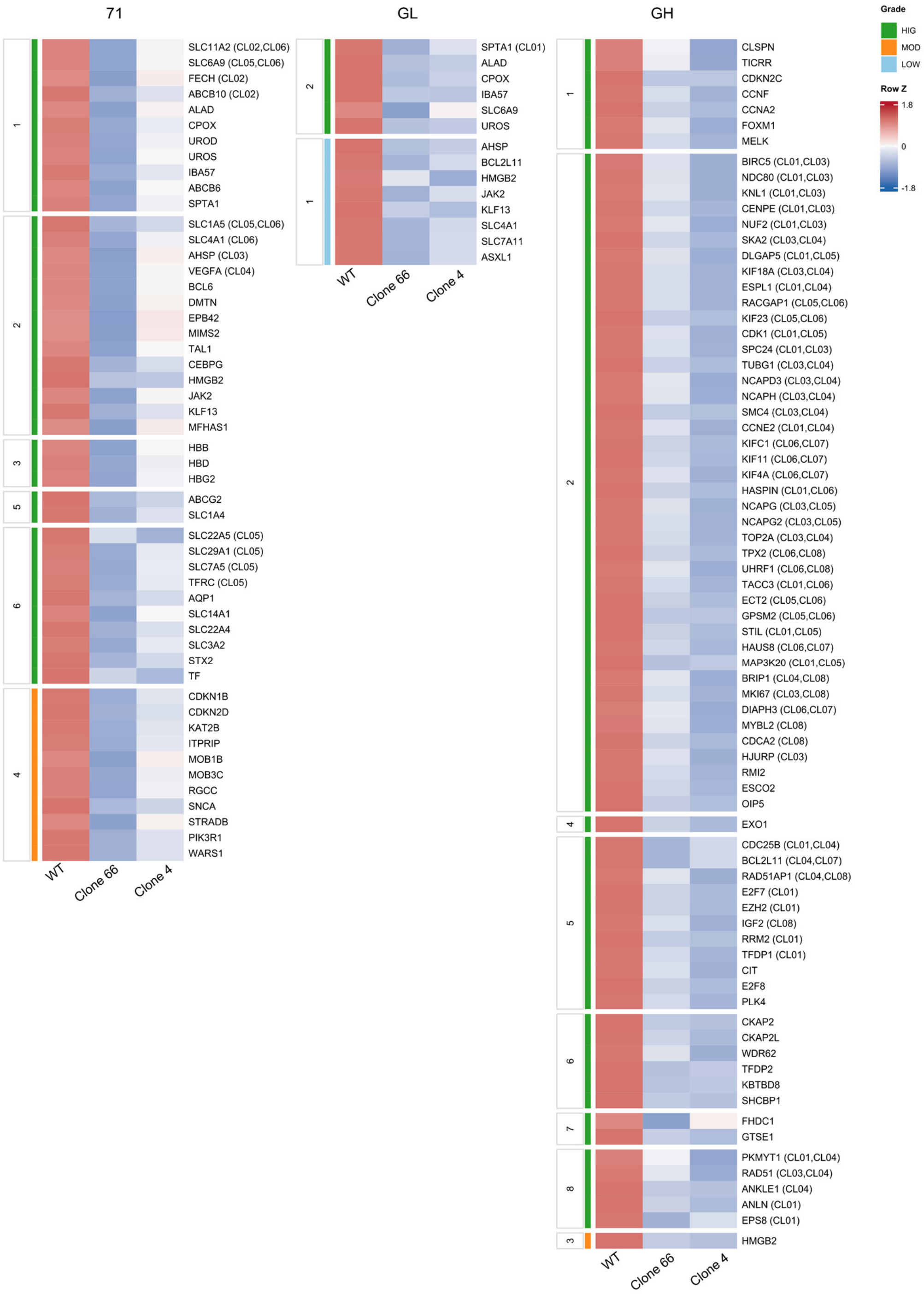
71
GL
GH
Grade
HIG
MOD
LOW
Row Z
1.8
0
-1.8
SLC11A2 (CL02,CL06)
SLC6A9 (CL05,CL06)
FECH (CL02)
ABCB10 (CL02)
ALAD
CPOX
UROD
UROS
IBA57
ABCB6
SPTA1
SLC1A5 (CL05,CL06)
SLC4A1 (CL06)
AHSP (CL03)
VEGFA (CL04)
BCL6
DMTN
EPB42
MIMS2
TAL1
CEBPG
HMGB2
JAK2
KLF13
MFHAS1
HBB
HBD
HBG2
ABCG2
SLC1A4
SLC22A5 (CL05)
SLC29A1 (CL05)
SLC7A5 (CL05)
TFRC (CL05)
AQP1
SLC14A1
SLC22A4
SLC3A2
STX2
TF
CDKN1B
CDKN2D
KAT2B
ITPRIP
MOB1B
MOB3C
RGCC
SNCA
STRADB
PIK3R1
WARS1
WT
Clone 66
Clone 4
SPTA1 (CL01)
ALAD
CPOX
IBA57
SLC6A9
UROS
AHSP
BCL2L11
HMGB2
JAK2
KLF13
SLC4A1
SLC7A11
ASXL1
CLSPN
TICRR
CDKN2C
CCNF
CCNA2
FOXM1
MELK
BIRC5 (CL01,CL03)
NDC80 (CL01,CL03)
KNL1 (CL01,CL03)
CENPE (CL01,CL03)
NUF2 (CL01,CL03)
SKA2 (CL03,CL04)
DLGAP5 (CL01,CL05)
KIF18A (CL03,CL04)
ESPL1 (CL01,CL04)
RACGAP1 (CL05,CL06)
KIF23 (CL05,CL06)
CDK1 (CL01,CL05)
SPC24 (CL01,CL03)
TUBG1 (CL03,CL04)
NCAPD3 (CL03,CL04)
NCAPH (CL03,CL04)
SMC4 (CL03,CL04)
CCNE2 (CL01,CL04)
KIFC1 (CL06,CL07)
KIF11 (CL06,CL07)
KIF4A (CL06,CL07)
HASPIN (CL01,CL06)
NCAPG (CL03,CL05)
NCAPG2 (CL03,CL05)
TOP2A (CL03,CL04)
TPX2 (CL06,CL08)
UHRF1 (CL06,CL08)
TACC3 (CL01,CL06)
ECT2 (CL05,CL06)
GPSM2 (CL05,CL06)
STIL (CL01,CL05)
HAUS8 (CL06,CL07)
MAP3K20 (CL01,CL05)
BRIP1 (CL04,CL08)
MKI67 (CL03,CL08)
DIAPH3 (CL06,CL07)
MYBL2 (CL08)
CDCA2 (CL08)
HJURP (CL03)
RMI2
ESCO2
OIP5
EXO1
CDC25B (CL01,CL04)
BCL2L11 (CL04,CL07)
RAD51AP1 (CL04,CL08)
E2F7 (CL01)
EZH2 (CL01)
IGF2 (CL08)
RRM2 (CL01)
TFDP1 (CL01)
CIT
E2F8
PLK4
CKAP2
CKAP2L
WDR62
TFDP2
KBTBD8
SHCBP1
FHDC1
GTSE1
PKMYT1 (CL01,CL04)
RAD51 (CL03,CL04)
ANKLE1 (CL04)
ANLN (CL01)
EPS8 (CL01)

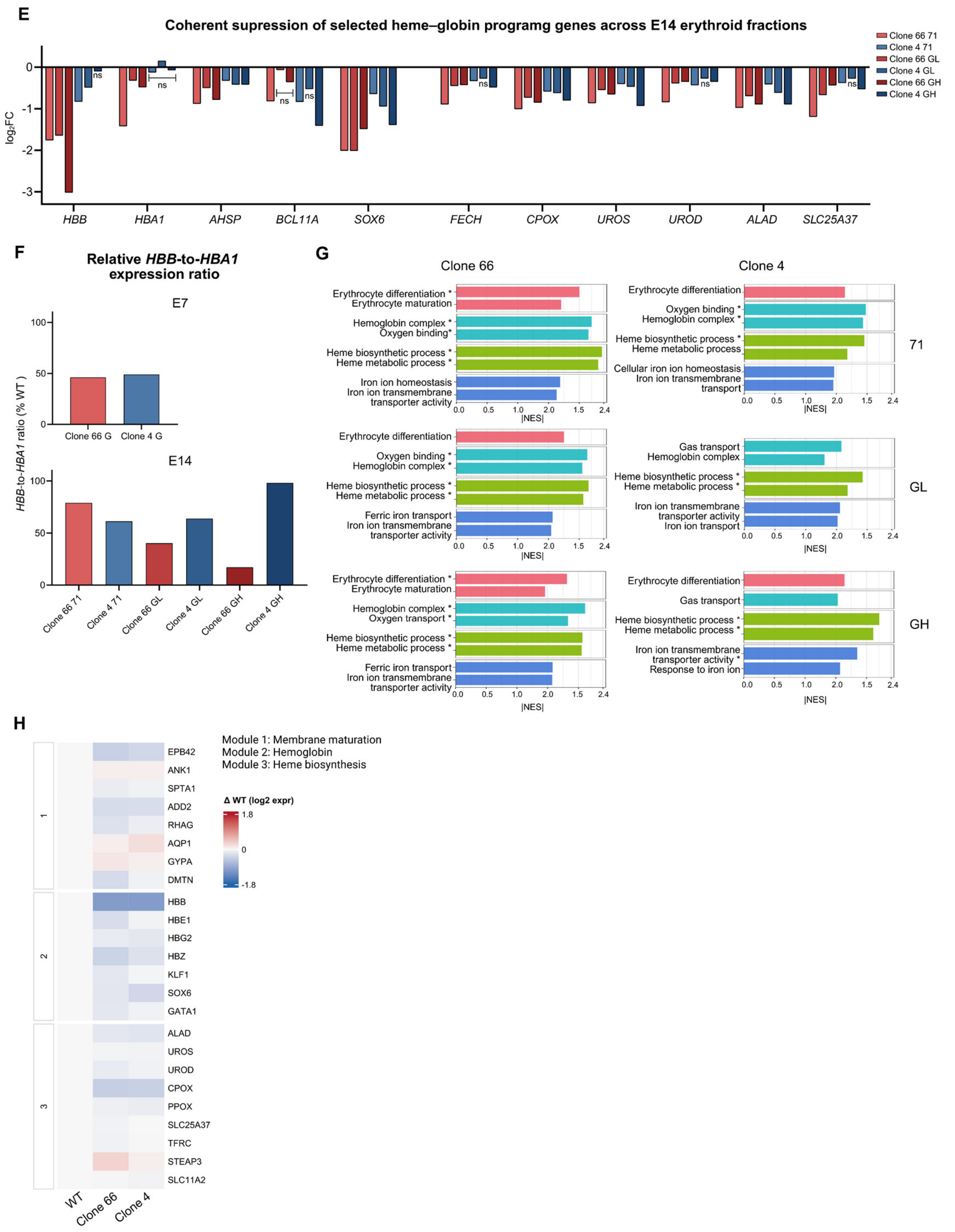

Figure S5. Supplementary bulk RNA sequencing (RNA-seq) data supporting the shared erythroid transcriptional defect. (A) Representative E7 sorting gate showing the CD71+GPA+ erythroid population collected for bulk RNA-seq. (B) Expression changes of *LINC01089* and its neighboring protein-coding genes (PCGs) in the E7 G fraction of Clone 66 and Clone 4 relative to WT. *LINC01089* remained reduced at E7, whereas the neighboring genes showed weaker or less consistent changes. (C)

Representative downregulated enrichment-map (EM) results displaying terms from the top three modules after redundancy reduction. Fractions are defined as CD71+GPA−/dim (71), CD71+GPAlow (GL), and CD71+GPAhigh (GH). In the 71 and GL fractions, both clones retained erythroid-associated terms, whereas GH was dominated by chromosome segregation-related terms. For better visualization, chromosome segregation- and mitosis-related terms were not displayed in the 71 and GL fractions of Clone 4 in this panel. The full Clone 4 results are provided in Supplementary Dataset S11. (D) Full heatmaps corresponding to Fig. 5D. Heatmaps are shown as row Z scores. Bridging genes in multiple clusters were assigned to the cluster with the lowest adjusted *p*-value, followed by a short list of their bridged clusters. The side color bar indicates gene-retention grade between the two clones by the 95% Jaccard overlap criterion: HIG (>0.25), MOD (0.20–0.25), LOW (<0.20). Cluster naming information is summarized in Table S8. (E) Expression changes in log2 fold change (log2FC) of representative heme-globin program genes across the E14 71, GL, and GH erythroid fractions in both clones relative to WT, showing predominantly downward shifts across fractions. Non-significant comparisons are labeled as ns where applicable. (F) Relative *HBB*-to-*HBA1* expression ratios in E7 and E14 erythroid populations of *LINC01089*+/− clones, calculated as $2^{(\mathrm{log2FC}\_\mathit{HBB} - \mathrm{log2FC}\_\mathit{HBA1})} \times 100\%$ relative to WT. Reduced ratios were observed in most erythroid populations, supporting impaired globin expression balance at the transcriptomic level. (G) Representative Gene Set Enrichment Analysis (GSEA) results of negatively enriched erythroid terms (FDR < 0.25) across the 71, GL, and GH fractions in both clones. Terms are grouped into: erythroid differentiation and maturation, hemoglobin, heme biosynthesis, and iron metabolism. Bars are shown as absolute normalized enrichment scores (|NES|). Terms with FDR < 0.05 are marked with asterisks (*). (H) ΔWT (log2 expression) heatmap showing broader projection of representative E14 defect-core genes onto the E7 data without strict adjusted *p*-value filtering. Genes are grouped into three modules representing membrane maturation, hemoglobin, and heme biosynthesis. Most genes showed a trend toward downregulation.

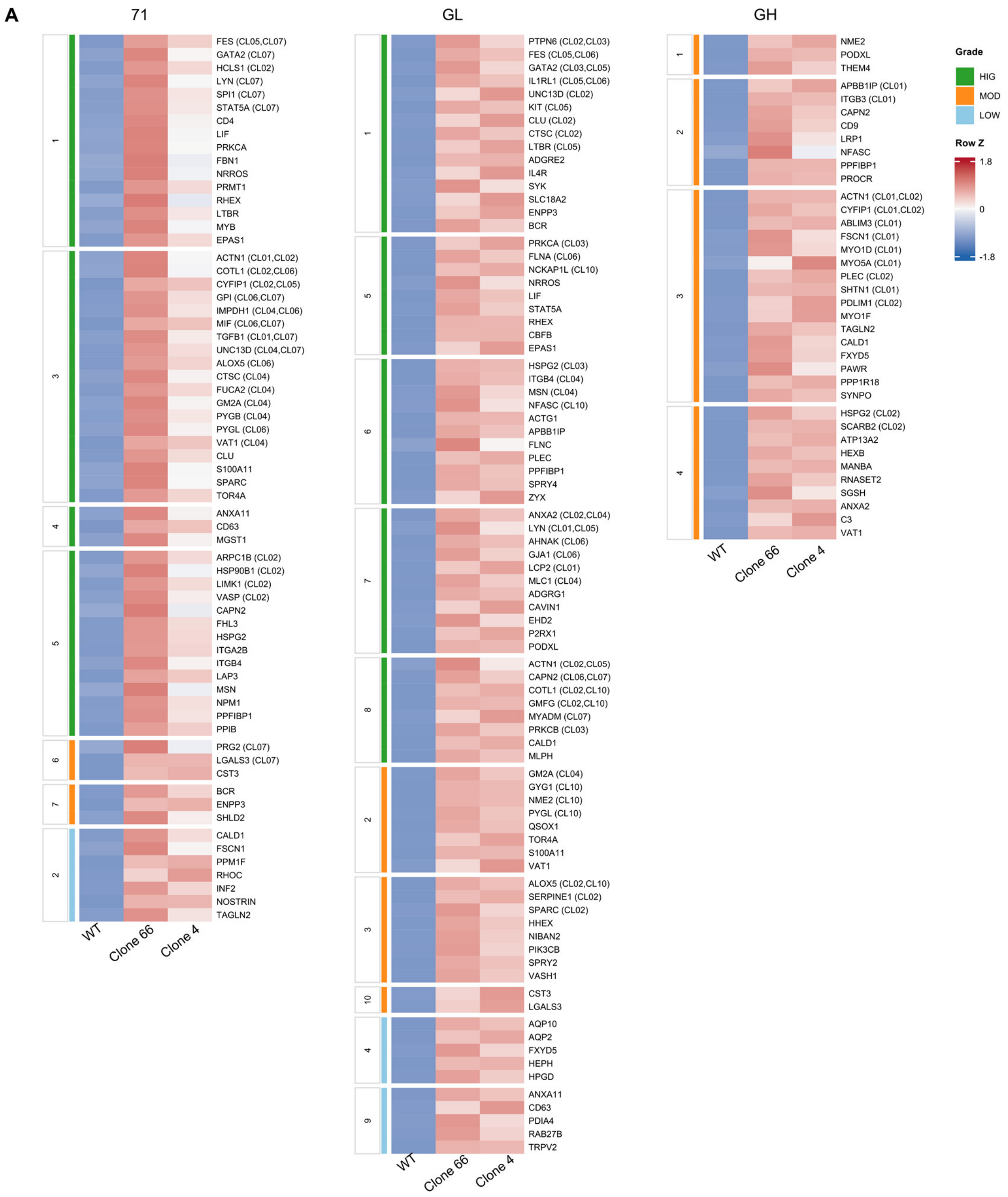
A
71
GL
GH
FES (CL05,CL07)
GATA2 (CL07)
HCLS1 (CL02)
LYN (CL07)
SPI1 (CL07)
STAT5A (CL07)
CD4
LIF
PRKCA
FBN1
NRROS
PRMT1
RHEX
LTBR
MYB
EPAS1
ACTN1 (CL01,CL02)
COTL1 (CL02,CL06)
CYFIP1 (CL02,CL05)
GPI (CL06,CL07)
IMPDH1 (CL04,CL06)
MIF (CL06,CL07)
TGFB1 (CL01,CL07)
UNC13D (CL04,CL07)
ALOX5 (CL06)
CTSC (CL04)
FUCA2 (CL04)
GM2A (CL04)
PYGB (CL04)
PYGL (CL06)
VAT1 (CL04)
CLU
S100A11
SPARC
TOR4A
ANXA11
CD63
MGST1
ARPC1B (CL02)
HSP90B1 (CL02)
LIMK1 (CL02)
VASP (CL02)
CAPN2
FHL3
HSPG2
ITGA2B
ITGB4
LAP3
MSN
NPM1
PPFIBP1
PPIB
PRG2 (CL07)
LGALS3 (CL07)
CST3
BCR
ENPP3
SHLD2
CALD1
FSCN1
PPM1F
RHOC
INF2
NOSTRIN
TAGLN2
WT
Clone 66
Clone 4
PTPN6 (CL02,CL03)
FES (CL05,CL06)
GATA2 (CL03,CL05)
IL1RL1 (CL05,CL06)
UNC13D (CL02)
KIT (CL05)
CLU (CL02)
CTSC (CL02)
LTBR (CL05)
ADGRE2
IL4R
SYK
SLC18A2
ENPP3
BCR
PRKCA (CL03)
FLNA (CL06)
NCKAP1L (CL10)
NRROS
LIF
STAT5A
RHEX
CBFB
EPAS1
HSPG2 (CL03)
ITGB4 (CL04)
MSN (CL04)
NFASC (CL10)
ACTG1
APBB1IP
FLNC
PLEC
PPFIBP1
SPRY4
ZYX
ANXA2 (CL02,CL04)
LYN (CL01,CL05)
AHNAK (CL06)
GJA1 (CL06)
LCP2 (CL01)
MLC1 (CL04)
ADGRG1
CAVIN1
EHD2
P2RX1
PODXL
ACTN1 (CL02,CL05)
CAPN2 (CL06,CL07)
COTL1 (CL02,CL10)
GMFG (CL02,CL10)
MYADM (CL07)
PRKCB (CL03)
CALD1
MLPH
GM2A (CL04)
GYG1 (CL10)
NME2 (CL10)
PYGL (CL10)
QSOX1
TOR4A
S100A11
VAT1
ALOX5 (CL02,CL10)
SERPINE1 (CL02)
SPARC (CL02)
HHEX
NIBAN2
PIK3CB
SPRY2
VASH1
CST3
LGALS3
AQP10
AQP2
FXYD5
HEPH
HPGD
ANXA11
CD63
PDIA4
RAB27B
TRPV2
NME2
PODXL
THEM4
APBB1IP (CL01)
ITGB3 (CL01)
CAPN2
CD9
LRP1
NFASC
PPFIBP1
PROCR
ACTN1 (CL01,CL02)
CYFIP1 (CL01,CL02)
ABLIM3 (CL01)
FSCN1 (CL01)
MYO1D (CL01)
MYO5A (CL01)
PLEC (CL02)
SHTN1 (CL01)
PDLIM1 (CL02)
MYO1F
TAGLN2
CALD1
FXYD5
PAWR
PPP1R18
SYNPO
HSPG2 (CL02)
SCARB2 (CL02)
ATP13A2
HEXB
MANBA
RNASET2
SGSH
ANXA2
C3
VAT1
Grade
HIG
MOD
LOW
Row Z
1.8
0
-1.8

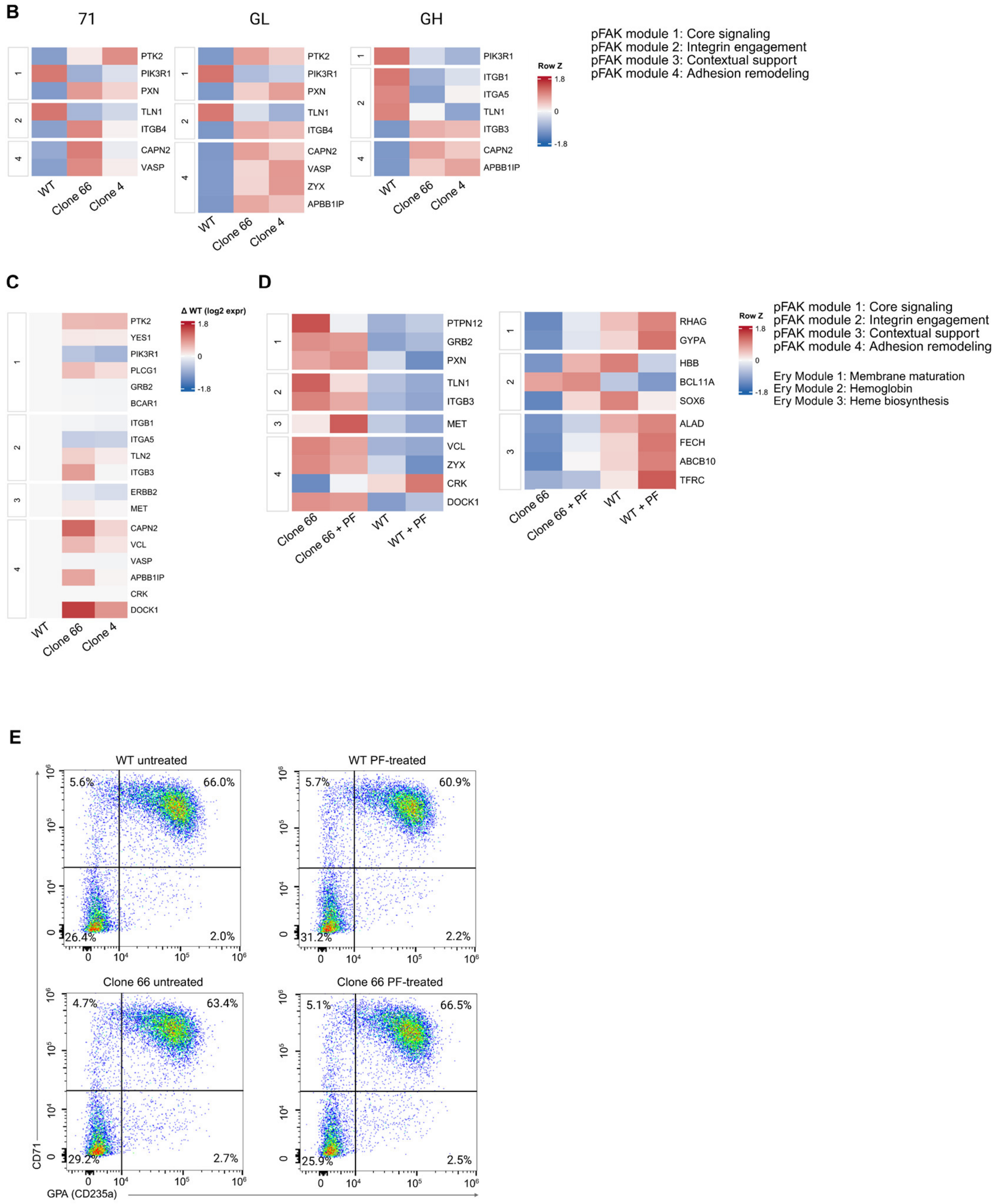

Figure S6. Supplementary analyses of focal adhesion signatures, phosphorylated focal adhesion kinase (pFAK)-proximal gene panels, and PF treatment responses. (A) Full retained-gene heatmaps of the shared upregulated modules across the 71, GL, and GH erythroid fractions at E14. Bridging genes in multiple clusters were assigned to the cluster with the lowest adjusted P value, followed by a short list of their bridged clusters. The side color bar indicates gene-retention grade between the two clones by the 95% Jaccard overlap criterion: HIG (>0.25), MOD (0.20–0.25), LOW (<0.20). Cluster naming information is summarized in Table S9. (B) Heatmaps of the

pFAK-proximal gene panel at E14. Genes were grouped into four pFAK-related modules representing core signaling, integrin engagement, contextual support (not retained after adjusted *p*-value filtering), and adhesion remodeling. (C) ΔWT (log2 expression) heatmap showing projection of the pFAK-proximal gene panel onto the E7 dataset. This broader projection without adjusted *p*-value filtering illustrates that the pFAK-related genes had already begun to shift at E7. (D) Row Z-score heatmaps showing PF-associated expression patterns in untreated and PF-treated Clone 66 and WT samples. These genes were filtered without an adjusted *p*-value cutoff but were required to show a consistent directional trend in Clone 66 + PF versus Clone 66 in the two batches. The selected genes showed PF-associated shifts in both the pFAK-proximal and erythroid panels. Notably, the hemoglobin module genes *HBB*, *BCL11A*, and *SOX6* showed opposite patterns in PF-treated WT samples compared to PF-treated Clone 66 samples. Left, pFAK-proximal panel results; right, erythroid panel results corresponding to membrane maturation, hemoglobin, and heme biosynthesis. (E) Representative CD71/GPA flow-cytometry plots from untreated and PF-treated Clone 66 and WT samples at E7. Changes in the CD71+GPA+ fraction are summarized in Fig. 6G.
